# Preparing the soil in knee osteoarthritis: a seed–systemic soil–local tissue soil framework for orthobiologic response heterogeneity

**DOI:** 10.3389/fbioe.2026.1946746

**Published:** 2026-09-08

**Authors:** Joelington Dias Batista, Ludimila Dias Silva, Jobson Dias Batista, Gabrielly Santos Pereira, Marcelo Silva

**Affiliations:** 1 Orthopain Pain Institute, Anápolis, Brazil; 2 Neuropsychobiology and Motor Behavior Laboratory, Faculty of Medicine of Ribeirão Preto, University of São Paulo, Ribeirão Preto, Brazil; 3 Laboratory of Neuroscience, Neuromodulation and Study of Pain (LANNeD), Federal University of Alfenas, Alfenas, Brazil

**Keywords:** adipose-derived therapies, knee osteoarthritis, orthobiologics, osteoarthritis, regenerative medicine

## Abstract

Musculoskeletal regenerative medicine has traditionally been guided by a product-centered model in which therapeutic outcomes are primarily attributed to the biological characteristics of the administered orthobiologic. However, the marked variability in clinical response suggests that treatment efficacy may also depend on the biological condition of the patient and the receptivity of the target tissue. This integrative review proposes a translational seed–soil framework in which therapeutic response is interpreted through the interaction among the biologic product (seed), systemic host biological readiness (systemic soil), and target tissue receptivity (local tissue soil), with knee osteoarthritis as the primary clinical and evidence context. A structured literature search was conducted in PubMed/MEDLINE, Embase, Scopus, and Web of Science, complemented by citation tracking. Evidence from clinical trials, systematic reviews, observational, imaging, biomarker, mechanistic, and conceptual studies was integrated according to prespecified framework domains. Our synthesis suggests that heterogeneity in orthobiologic outcomes can be more coherently interpreted when three interacting biological layers are considered together: product composition and potency, systemic host factors, and local joint and tissue characteristics. Although several orthobiologic classes demonstrate meaningful clinical signals in knee osteoarthritis, treatment effects remain inconsistent because of variability in product processing and composition, contextual and placebo effects, the strength of active comparators, differences in pain phenotypes, and the multicomponent nature of osteoarthritic joint disease. Importantly, symptomatic improvement should be distinguished from structural regeneration, which remains inconsistently demonstrated across orthobiologic studies. Rigorous product characterization remains essential, but the proposed seed–soil framework extends this perspective by integrating product characteristics with systemic host biology and local tissue receptivity. Although optimization of systemic and local soil conditions has not yet been definitively validated in randomized trials, the proposed framework provides a biologically grounded structure for explaining response heterogeneity, improving patient stratification, and guiding the design of future translational and clinical studies.

## 1 Introduction

Musculoskeletal regenerative medicine has developed around a deceptively simple clinical question: which biological product should be administered? This product-centered perspective was historically understandable, as early translational research focused primarily on determining whether orthobiologic interventions such as platelet-rich plasma (PRP), bone marrow aspirate and bone marrow aspirate concentrate, mesenchymal stromal cells (MSCs), and adipose-derived preparations could reduce symptoms or promote tissue repair in chronic musculoskeletal disorders. This approach contributed substantially to the development of the field and enabled the emergence of several promising therapeutic strategies. However, the persistence of highly variable outcomes across clinical trials and real-world practice indicates that product identity alone cannot fully explain therapeutic response (Navani et al., 2025; Andia et al., 2025).

Knee osteoarthritis (OA) illustrates this limitation with particular clarity. Traditionally described as a degenerative disorder characterized by progressive cartilage loss, OA is now understood as a heterogeneous, multifactorial, and whole-joint disease. Its pathophysiology involves not only articular cartilage but also subchondral bone, synovium, menisci, ligaments, periarticular muscles, sensory structures, and local adipose tissues. Mechanical overload, low-grade inflammation, metabolic dysfunction, previous joint trauma, aging-related changes, and altered neuromuscular control may interact differently among patients, producing distinct structural, inflammatory, metabolic, and pain phenotypes (Tang et al., 2025). Consequently, patients with similar radiographic disease severity may experience markedly different symptoms, rates of progression, and responses to treatment.

This biological and clinical heterogeneity has important implications for regenerative interventions. An intra-articular product is not delivered into a passive or biologically uniform environment. Instead, it is introduced into a complex joint ecosystem that may differ substantially in synovial inflammation, cartilage integrity, subchondral bone remodeling, meniscal damage, mechanical alignment, adipose tissue activity, vascular supply, cellular senescence, and nociceptive sensitization. These local characteristics may influence cell survival, paracrine signaling, growth-factor activity, extracellular matrix turnover, and the capacity of resident tissues to respond to regenerative stimuli. Therefore, the same orthobiologic preparation may produce different effects depending on the biological receptivity of the target joint (Tang et al., 2025; Navani et al., 2025).

A parallel conceptual shift has occurred regarding the biological products themselves. PRP, for example, is increasingly recognized not as a single standardized therapy but as a spectrum of autologous preparations that differ in platelet concentration, leukocyte content, red blood cell contamination, activation method, processing technique, injection volume, and dosing schedule (Andia et al., 2025). Similar variability exists among bone marrow-derived and adipose-derived therapies, including differences in tissue harvesting, centrifugation or processing protocols, cellular composition, stromal vascular components, culture conditions, viability, potency, and regulatory classification (Batista et al., 2026; Navani et al., 2025). This product heterogeneity complicates comparisons across studies and limits the assumption that interventions sharing the same broad label are biologically equivalent.

Despite the importance of product characteristics, growing evidence suggests that treatment response may also depend on the systemic biological condition of the recipient. Factors such as age, obesity, metabolic syndrome, insulin resistance, chronic inflammation, nutritional status, sleep quality, physical inactivity, medication use, smoking, psychological distress, and comorbid disease may influence immune regulation, cellular function, vascular responses, and tissue repair capacity. These systemic factors can modify both the composition of autologous biological products and the ability of host tissues to respond after administration. Accordingly, regenerative outcomes may reflect not only what is injected, but also the biological readiness of the patient receiving the intervention (Lana et al., 2024; Navani et al., 2025).

Host-oriented proposals such as the SDIMMMER framework have therefore emphasized the potential relevance of sleep, diet, interactions with the microbiome, metabolism, medications, laboratory evaluation, mental health, exercise, and rehabilitation before and after regenerative procedures (Lana et al., 2024). Although the direct clinical benefit of systematically optimizing all these domains has not yet been definitively established in randomized trials, the framework reflects an important transition from isolated product selection toward a broader evaluation of host biology. Within this perspective, systemic optimization should not be interpreted as a substitute for an effective orthobiologic product, but rather as a potential modifier of biological responsiveness (Lana et al., 2024; Navani et al., 2025).

The systemic-soil component proposed here intentionally overlaps with the host-oriented domains incorporated in the SDIMMMER framework, and these host factors are not presented as novel. The distinct contribution of the present framework is to place systemic host biology within a three-layer model that explicitly separates the biologic intervention (“seed”), the systemic host environment (“systemic soil”), and the structural, inflammatory, mechanical, and sensory characteristics of the target joint (“local tissue soil”). In addition, whereas SDIMMMER primarily emphasizes optimization of host physiology around regenerative procedures, the present framework is intended principally as an explanatory and hypothesis-generating model of treatment-response heterogeneity rather than as a bundled optimization protocol.

The local joint environment represents a second and distinct level of biological readiness. Even when systemic conditions are favorable, advanced structural disruption, persistent synovitis, severe malalignment, extensive meniscal damage, subchondral bone abnormalities, or centrally mediated pain may limit the clinical effectiveness of an intra-articular intervention. Conversely, patients with preserved tissue architecture, modifiable inflammatory activity, less advanced disease, and predominantly peripheral nociceptive pain may provide a more receptive biological environment. The distinction between systemic host readiness and local tissue receptivity is therefore essential, because these dimensions may influence therapeutic outcomes through different mechanisms and may require different assessment and management approaches (Tang et al., 2025; Navani et al., 2025).

This distinction is particularly relevant when interpreting evidence from adipose-derived intra-articular therapies. A 2026 systematic review including 19 randomized controlled trials identified meaningful clinical signals for pain reduction, functional improvement, and short-to medium-term safety (Batista et al., 2026). However, the review also demonstrated substantial heterogeneity in processing methods, cellular composition, comparators, injection protocols, patient characteristics, outcome measures, and follow-up periods. Moreover, although symptomatic improvements were reported in several studies, consistent structural cartilage regeneration was not uniformly demonstrated (Batista et al., 2026). These findings suggest that clinical benefit may arise through anti-inflammatory, immunomodulatory, trophic, analgesic, or joint-homeostatic mechanisms rather than through predictable anatomical regeneration alone.

The interpretation of orthobiologic effectiveness is further complicated by contextual effects and the strength of comparison groups. Intra-articular procedures may be associated with substantial nonspecific effects related to treatment expectations, clinical interaction, needle-based intervention, aspiration of joint fluid, and natural symptom fluctuation. In addition, comparators such as corticosteroids, hyaluronic acid, saline injections, rehabilitation programs, or other active orthobiologics may themselves produce meaningful improvements. Consequently, small between-group differences do not necessarily indicate biological inactivity, while large within-group changes cannot automatically be attributed to regenerative mechanisms (Batista et al., 2026; Andia et al., 2025). These methodological considerations reinforce the need for frameworks capable of integrating product properties with patient- and tissue-level determinants of response.

A predominantly product-centered model alone may be insufficient to explain why apparently similar interventions produce substantial benefit in some patients, modest or transient improvement in others, and no meaningful response in a third group. Product characterization remains indispensable and, in the proposed framework, is integrated with systemic host biology and the biological condition of the target tissue.

In the framework proposed here, the “seed” represents the biological product, including its composition, processing, viability, potency, dose, and delivery characteristics. The “systemic soil” represents the biological readiness of the host, encompassing metabolic, inflammatory, nutritional, behavioral, medication-related, and rehabilitation-related factors. The “local tissue soil” represents the structural, cellular, inflammatory, mechanical, vascular, and nociceptive state of the target joint. These three components should not be viewed as independent variables, but as interacting biological layers that collectively shape treatment response. Throughout this review, “preparing the soil” is used as a conceptual metaphor and should not be interpreted as implying that pre-treatment modification of systemic or local factors has been demonstrated to enhance orthobiologic efficacy.

Importantly, the proposed framework does not claim that systematic optimization of systemic and local soil conditions has already been proven to improve orthobiologic outcomes. Rather, it provides a biologically grounded structure for interpreting response heterogeneity, identifying potentially modifiable determinants, and designing more informative clinical studies. Future trials may benefit from moving beyond broad diagnostic categories and evaluating whether baseline metabolic status, inflammatory phenotype, pain mechanism, joint structure, tissue quality, and product characteristics interact to determine therapeutic response.

Accordingly, the primary aim of this integrative review was to propose an explanatory and hypothesis-generating framework for organizing potential determinants of orthobiologic response heterogeneity in knee OA. The framework is not presented as a validated clinical algorithm or prescriptive trial-design standard. A secondary aim was to identify measurable variables and testable interactions that may inform future prospective studies.

## 2 Methods

### 2.1 Study design

This study was conducted as an integrative review and conceptual framework synthesis guided by the methodological approach proposed by Whittemore and Knafl (2005). The review process comprised five interconnected stages: problem identification, literature search, data evaluation, data analysis, and presentation of findings. This approach was selected because the research question required integration of heterogeneous forms of evidence, including randomized and non-randomized clinical studies, systematic reviews, observational studies, imaging and biomarker investigations, mechanistic studies, and conceptual publications.

The primary purpose of the review was explanatory and hypothesis-generating. It was designed to organize potential determinants of heterogeneity in orthobiologic treatment response rather than to estimate a pooled treatment effect, establish comparative efficacy between interventions, formulate clinical practice recommendations, or validate a treatment-selection algorithm.

Knee osteoarthritis (OA) was selected as the primary disease context because of its heterogeneous pathophysiology, whole-joint involvement, diversity of pain and structural phenotypes, and extensive literature on intra-articular orthobiologic interventions. The review was developed to examine whether the available evidence could be organized within a three-layer framework comprising the characteristics of the biologic intervention (“seed”), systemic host biology (“systemic soil”), and the structural, inflammatory, mechanical, and sensory characteristics of the target joint (“local tissue soil”).

No single publication was used to determine eligibility, evidence weighting, or framework construction. Previously published systematic reviews, including Batista et al. (2026) for adipose-derived intra-articular therapies, were treated as domain-specific evidence sources and considered alongside independent systematic reviews, randomized trials, observational studies, imaging and biomarker research, and relevant mechanistic and conceptual literature.

### 2.2 Conceptual scope and eligibility criteria

Eligibility criteria were operationalized using a Population–Concept–Context (PCC) structure. The population comprised adults with knee OA for clinical, interventional, observational, imaging, and biomarker evidence. The concept encompassed factors potentially associated with heterogeneity in response to orthobiologic interventions, including characteristics of the biologic product (“seed”), systemic host factors (“systemic soil”), local joint and tissue characteristics (“local tissue soil”), pain phenotype, imaging and biochemical biomarkers, contextual effects, comparator characteristics, and patient-stratification variables. The context was the evaluation and use of intra-articular orthobiologic interventions in knee OA.

Clinical and interventional studies were eligible when they included adults with knee OA and evaluated an intra-articular orthobiologic intervention or investigated patient-, product-, tissue-, imaging-, biomarker-, or pain-related characteristics potentially relevant to treatment response. Eligible orthobiologic interventions included platelet-rich plasma (PRP), bone marrow aspirate (BMA), bone marrow aspirate concentrate (BMAC), mesenchymal stromal cell-based products, adipose-derived stromal cells (ADSCs), stromal vascular fraction (SVF), microfragmented adipose tissue (MFAT), and related autologous or allogeneic biologic preparations.

Systemic-host evidence was eligible when it addressed prespecified factors potentially relevant to biological responsiveness or treatment heterogeneity, including metabolic health, obesity or body composition, insulin resistance, systemic inflammation, nutritional status, sleep and chronobiology, medication exposure, physical activity or conditioning, rehabilitation capacity, smoking, psychological factors, microbiome-related factors, and clinically relevant comorbidities.

Local-tissue evidence was eligible when it addressed structural disease severity, cartilage integrity, synovitis or effusion, meniscal abnormalities, subchondral bone changes, bone marrow lesions, mechanical alignment, periarticular muscle function, local adipose-tissue characteristics, vascular characteristics, nociceptive mechanisms, neuropathic-like pain, pain sensitization, or central sensitization. Imaging and biomarker studies were eligible when they evaluated variables with potential prognostic, predictive, phenotypic, mechanistic, or treatment-stratification relevance.

Eligible publication types included randomized controlled trials, non-randomized controlled clinical studies, prospective and retrospective observational studies, systematic reviews and meta-analyses, evidence-based clinical guidelines, consensus statements, state-of-the-art and narrative reviews, and human imaging or biomarker studies. Conceptual publications were considered when they directly contributed to the definition, historical origin, or interpretation of a prespecified framework domain.

The primary electronic search was restricted to English-language publications published from January 2016 through June 2026. This interval was selected to capture contemporary developments in orthobiologic characterization, precision medicine, imaging, biomarkers, and intra-articular regenerative approaches. Earlier seminal publications were considered only through targeted backward citation tracking when necessary to establish the historical origin, theoretical foundation, or biological basis of a key framework construct. No unrestricted electronic search of the pre-2016 literature was performed.

Human studies constituted the primary evidence base. Selected preclinical animal or *in vitro* studies were considered only when identified through citation tracking and when they provided mechanistic information directly relevant to a prespecified framework component that could not be adequately characterized from human evidence alone. Such evidence was used exclusively to support biological plausibility and was not interpreted as evidence of clinical effectiveness, treatment-response modification, prognostic performance, or patient stratification in humans.

Clinical studies involving musculoskeletal disorders other than knee OA were excluded from the primary clinical evidence base. Non-knee-OA publications could be considered only when they established a general conceptual or mechanistic principle directly relevant to a prespecified framework domain; they were not used to infer clinical effectiveness or treatment response in knee OA.

Conference abstracts, meeting proceedings without a full peer-reviewed publication, study protocols without outcome data, editorials, letters without original data or substantive evidence synthesis, and publications for which the full text was unavailable were excluded.

Publications were excluded when they: (1) did not address knee OA and did not provide a directly relevant conceptual or mechanistic contribution to a prespecified framework domain; (2) focused exclusively on technical product preparation or laboratory procedures without implications for biological characteristics, clinical response, or response heterogeneity; (3) did not address any of the prespecified seed, systemic-soil, local-tissue-soil, pain, imaging, biomarker, contextual-effect, comparator, or stratification domains; (4) consisted exclusively of preclinical evidence without a direct mechanistic relationship to the framework; (5) duplicated data reported in another publication without providing additional relevant information; (6) were conference abstracts or other non-full-text reports; or (7) consisted primarily of unsupported expert opinion without a substantive evidence-based or conceptual contribution.

Eligibility and evidentiary weighting were treated as distinct processes. Publications meeting the eligibility criteria were not excluded solely on the basis of study design. Methodological design, directness, consistency, and strength of evidence were instead considered during the subsequent data-evaluation and synthesis stages.

Local-tissue evidence was eligible when it addressed structural disease severity, cartilage integrity, synovitis or effusion, meniscal abnormalities, subchondral bone changes, bone marrow lesions, mechanical alignment, periarticular muscle structure or function, local adipose-tissue characteristics, vascular characteristics, or peripheral nociceptive mechanisms directly related to joint pathology. Pain-related evidence, including neuropathic-like symptoms, catastrophizing, pain sensitization, and central sensitization-related features, was treated as a cross-cutting domain because these variables may reflect interactions among peripheral joint pathology, central nervous system processing, and psychosocial factors.

### 2.3 Information sources and literature search

A structured literature search was conducted in PubMed/MEDLINE, Embase, Scopus, and Web of Science Core Collection. The primary electronic search covered publications from January 2016 through June 2026 and was restricted to English-language publications.

The electronic search was supplemented by backward citation tracking of eligible systematic reviews, state-of-the-art reviews, conceptual publications, and other highly relevant sources. Forward citation tracking was used selectively to identify subsequent publications directly related to key framework constructs. Citation tracking was also used to identify seminal publications predating 2016 and selected mechanistic preclinical studies meeting the exceptions defined in Section 2.2.

To provide broad but prespecified coverage without relying on a single excessively restrictive query, the electronic search was organized into five complementary thematic domains corresponding to the review objectives.

The first domain addressed knee OA and orthobiologic products (“seed”), including PRP, BMA, BMAC, mesenchymal stromal cell-based therapies, ADSCs, SVF, MFAT, and related regenerative or cell- and tissue-based intra-articular interventions.

The second domain addressed systemic host biology (“systemic soil”), including metabolic health, obesity and body composition, insulin resistance, systemic inflammation, nutrition, sleep and chronobiology, medications, physical activity, rehabilitation, smoking, psychological factors, comorbidities, and microbiome-related factors.

The third domain addressed local joint and tissue biology (“local tissue soil”), including cartilage, synovitis and effusion, subchondral bone, bone marrow lesions, meniscal pathology, mechanical alignment, infrapatellar adipose tissue, periarticular muscle, nociceptive mechanisms, neuropathic-like pain, and peripheral or central pain sensitization.

The fourth domain addressed imaging, biomarkers, endotypes, and patient stratification, including MRI-based features, biochemical and synovial-fluid biomarkers, phenotypes and molecular endotypes, prognostic and predictive factors, treatment response, responder classification, precision medicine, and treatment-effect modification.

The fifth domain addressed contextual effects and comparator characteristics, including placebo and contextual responses, saline or sham procedures, active comparators, corticosteroids, hyaluronic acid, PRP and other orthobiologics, rehabilitation, and the distinction between within-group clinical improvement and incremental between-group treatment effects.

Controlled vocabulary terms, when available, were combined with free-text keywords and synonyms. Terms within individual conceptual blocks were combined using OR, whereas distinct conceptual blocks were combined using AND. Syntax and indexing terms were adapted to the requirements of each database.

The complete database-specific search strategies, including controlled vocabulary, free-text terms, Boolean operators, search limits, and date restrictions, are provided in Supplementary Table S1.

The primary database search was designed to identify the contemporary human evidence base and did not constitute a systematic search of the entire preclinical literature or of publications predating 2016. Seminal pre-2016 publications and selected preclinical studies identified through citation tracking were therefore classified separately from records retrieved through the primary electronic search.

### 2.4 Literature identification and selection

Records retrieved from the five complementary thematic searches were combined within each database and internally deduplicated. Results from the four databases were subsequently merged and duplicates across databases were removed before eligibility screening.

Two reviewers (G.S.P. and M.L.S.) independently screened titles and abstracts and subsequently assessed potentially eligible publications in full text. Disagreements regarding eligibility were resolved through discussion and consensus; when consensus could not be reached, a third reviewer (J.D.B.) adjudicated the decision.

Screening was conducted in two sequential stages. Titles and abstracts were first assessed against the eligibility criteria defined in Section 2.2. Publications considered potentially relevant then underwent full-text assessment. Reasons for full-text exclusion were based on the predefined population, conceptual-domain, publication-type, language, date, and evidence-source criteria.

Additional publications identified through backward or forward citation tracking were subjected to the same conceptual relevance assessment. Seminal pre-2016 publications and selected preclinical studies were retained only when they met the specific exceptions defined in Section 2.2 and were classified separately from the contemporary human evidence base.

The breadth of literature identification was explicitly distinguished from evidentiary weighting. Database retrieval was designed to capture evidence across the prespecified framework domains, whereas methodological design, directness, consistency, replication, and relevance to treatment-response heterogeneity informed the interpretive weight assigned during synthesis.

The number of records retrieved from each database, duplicates removed, unique records screened, full-text publications assessed, publications retained from the primary electronic search, and additional publications incorporated through citation tracking are reported in Supplementary Table S2.

### 2.5 Data extraction and evidence mapping

Relevant information was extracted using a structured evidence-mapping approach aligned with the objectives and prespecified domains of the review. Extracted information included publication type, study population, intervention or exposure, orthobiologic class when applicable, comparator characteristics, principal clinical outcomes, structural or imaging outcomes, biomarker findings, safety information, proposed biological mechanisms, host-related variables, local tissue characteristics, pain phenotype, and potential implications for prognostic assessment, treatment-response heterogeneity, or patient stratification.

For orthobiologic interventions, particular attention was given to characteristics capable of defining the biological and technical identity of the seed, including tissue source, harvest conditions, processing method, cellular or molecular composition, platelet and leukocyte characteristics when applicable, product viability, dose, injection volume, number and schedule of administrations, delivery method, and comparator.

Systemic variables were mapped to the systemic-soil domain and included age, obesity and body composition, metabolic dysfunction, insulin resistance, systemic inflammatory status, nutrition, sleep, physical activity or conditioning, medication exposure, smoking, psychological factors, microbiome-related variables, comorbidities, and rehabilitation capacity.

Local variables were mapped to the local-tissue-soil domain and included radiographic and structural severity, cartilage integrity, synovitis and effusion, meniscal pathology, subchondral bone abnormalities, bone marrow lesions, mechanical alignment, periarticular muscle structure and function, local adipose-tissue characteristics, vascular features, and peripheral nociceptive mechanisms directly related to joint pathology. Pain phenotype, including neuropathic-like symptoms, catastrophizing, and central sensitization-related features, was mapped as a cross-cutting modifier. Rehabilitation capacity, adherence, and broader physical conditioning were similarly treated as cross-cutting functional or host-level variables.

Comparator type and information relevant to contextual or nonspecific procedural effects were additionally mapped as prespecified cross-cutting variables. Particular attention was given to whether reported improvement represented within-group change, comparison with an inert or minimally active control, or incremental benefit over an active comparator.

### 2.6 Methodological appraisal and evidence weighting

The data-evaluation stage followed the principle that heterogeneous evidence sources should not be interpreted as methodologically equivalent. Because the review incorporated systematic reviews, randomized and non-randomized clinical studies, observational research, imaging and biomarker investigations, mechanistic studies, and conceptual publications, no single risk-of-bias instrument was applicable across the complete evidence base.

Sources were therefore appraised according to a structured set of interpretive dimensions comprising: methodological design, directness to knee OA and the specific framework question, adequacy of the comparison group when applicable, consistency with other evidence, replication, and relevance to treatment-response heterogeneity. For clinical-effect claims, greater interpretive weight was assigned to systematic reviews and meta-analyses, randomized controlled trials, controlled clinical evidence, and replicated human findings. Observational, imaging, and biomarker studies were weighted primarily according to their ability to characterize associations, phenotypes, prognostic factors, or candidate treatment-response modifiers.

When systematic reviews or meta-analyses reported formal risk-of-bias or certainty-of-evidence assessments, these judgments were considered when interpreting their conclusions. Primary studies were interpreted according to their design and reported methodological limitations. No numerical quality score was generated across different evidence types, because such a score would imply comparability among fundamentally different methodological designs.

Conceptual publications and selected preclinical studies were not used as substitutes for clinical evidence. Their contribution was restricted to theoretical development, historical grounding, or mechanistic plausibility. Similarly, no individual systematic review was designated as an overarching evidentiary anchor. The systematic review by Batista et al. (2026), for example, was used specifically to inform the adipose-derived therapy domain and was interpreted alongside independent systematic reviews, meta-analyses, and randomized trials addressing related and complementary orthobiologic questions.

This appraisal strategy was intended to guide interpretation rather than to provide a formal GRADE-based certainty rating for the complete integrative evidence base.

### 2.7 Framework construction, data synthesis, and interpretive classification

Evidence was synthesized narratively and conceptually according to the prespecified domains of the review. An initial three-domain scaffold—seed, systemic soil, and local tissue soil—was specified from the conceptual objective of the review rather than derived as an empirical outcome of the literature search. Evidence mapping was subsequently used to refine the operational definition of each domain, characterize relationships among them, and identify cross-cutting variables relevant to treatment-response heterogeneity.

The seed domain incorporated the biological and technical characteristics of the administered orthobiologic product. The systemic-soil domain incorporated host-level physiological, metabolic, inflammatory, behavioral, medication-related, and rehabilitative characteristics potentially relevant to biological responsiveness. The local-tissue-soil domain incorporated structural, inflammatory, mechanical, cellular, vascular, and sensory characteristics of the target joint.

Pain phenotype, imaging, biomarkers, contextual effects, comparator characteristics, rehabilitation, and patient stratification were treated as prespecified cross-cutting domains and mapped according to their relationship with one or more of the three principal components.

The synthesis examined recurring patterns, areas of convergence or inconsistency, and potential relationships among product-, host-, and tissue-level variables. Interpretation was calibrated according to methodological directness and evidentiary strength as described in Section 2.6.

To make the epistemic status of the synthesis explicit, statements were classified into three interpretive categories. Direct evidence-supported observations comprised conclusions supported by systematic reviews or meta-analyses, randomized or controlled clinical evidence, or replicated human findings directly relevant to knee OA. Translationally supported inferences comprised interpretations supported primarily by observational, imaging, biomarker, or mechanistic evidence but not prospectively validated as modifiers of orthobiologic treatment response. Hypothesis-generating propositions comprised conceptual extensions arising from integration of the available evidence for which direct prospective validation was lacking. The evidence-status classification assigned to the principal framework domains and cross-cutting propositions is summarized in Supplementary Table S3.

Empirically supported findings were distinguished from interpretive synthesis, whereas translational inferences and conceptual propositions were expressed using appropriately cautious language.

In particular, the synthesis did not assume that modification or optimization of systemic- or local-soil variables has been demonstrated to improve orthobiologic outcomes. In the absence of direct treatment-effect-modification evidence, these variables were interpreted as candidate moderators, mediators, prognostic indicators, or stratification factors requiring prospective validation.

For interpretive purposes, associations, prognostic factors, treatment-effect modifiers, mediators, and causal effects of factor modification were considered conceptually distinct. An association was defined as a relationship between a variable and an outcome without implying predictive or causal relevance. A prognostic factor was considered a variable associated with outcome irrespective of treatment assignment. A treatment-effect modifier required evidence that the relative effect of an intervention differed across levels of the variable, ideally through a prespecified treatment-by-variable interaction. A mediator was considered a variable potentially located on the causal pathway between intervention and outcome. Evidence that modification of a soil factor causally improves orthobiologic efficacy required prospective interventional evidence and was not inferred from association or prognostic studies alone.

Accordingly, the resulting Seed–Systemic Soil–Local Tissue Soil framework was developed as an explanatory and hypothesis-generating model for organizing potential determinants of orthobiologic response heterogeneity in knee OA. It was not intended to function as a validated clinical treatment algorithm, a prescriptive patient-selection strategy, or a formal methodological standard for clinical trial design.

## 3 Results

### 3.1 Integrative synthesis and framework development

Using the prespecified three-domain scaffold, the available evidence was organized according to characteristics of the biologic product (“seed”), systemic host biology (“systemic soil”), and local joint and tissue characteristics (“local tissue soil”). Cross-cutting evidence concerning pain phenotype, functional loading, rehabilitation, contextual effects, comparator characteristics, imaging, and biochemical or molecular biomarkers was mapped across these domains. The evidentiary status of the principal observations, translational inferences, and hypothesis-generating propositions supporting the framework is summarized in Supplementary Table S3.

The resulting Seed–Systemic Soil–Local Tissue Soil framework was developed as an explanatory and hypothesis-generating model. It does not assume that optimization of systemic or local soil conditions has already been demonstrated to improve orthobiologic outcomes in prospective randomized trials. Instead, it organizes current clinical and mechanistic evidence into biologically plausible, partly measurable, and potentially modifiable domains that may explain why apparently similar interventions produce markedly different outcomes among patients sharing the same clinical diagnosis.

**FIGURE 1:**
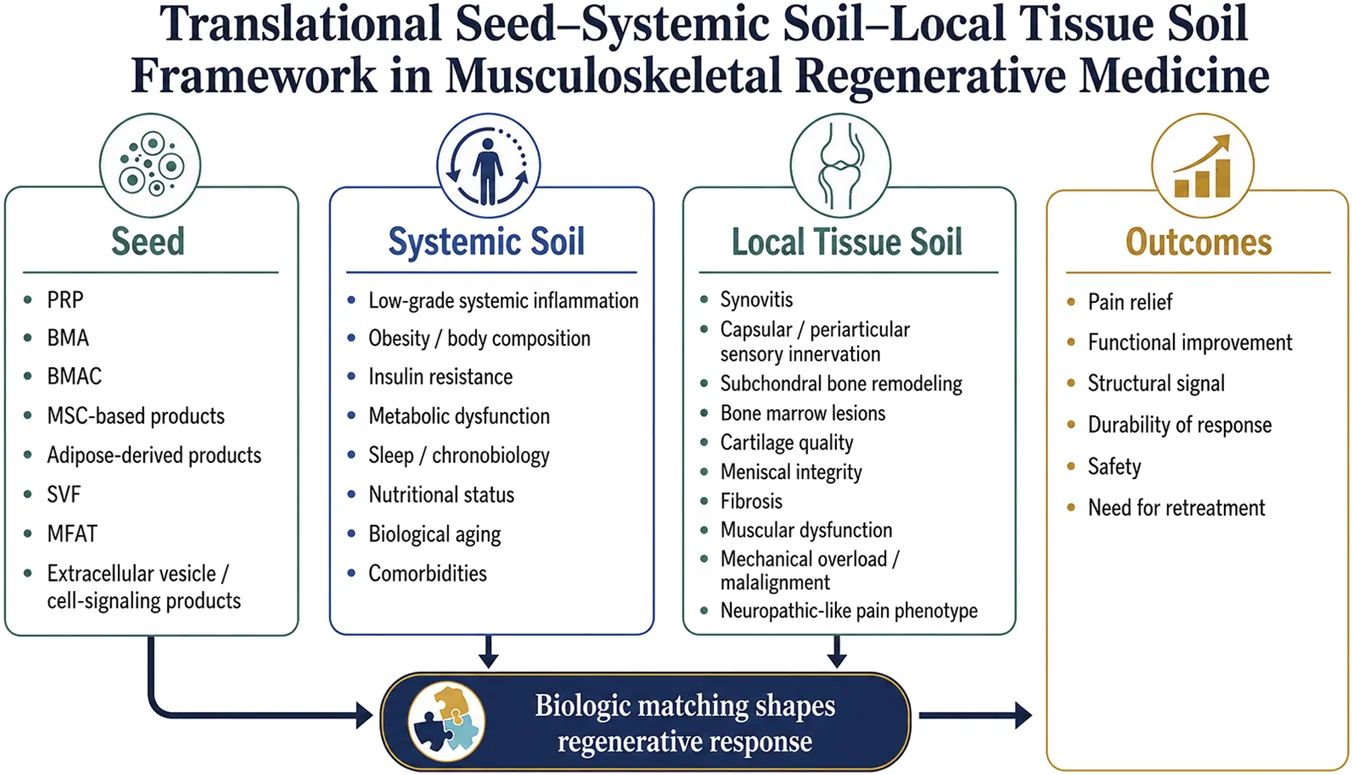
Seed–Systemic Soil–Local Tissue Soil framework in knee osteoarthritis. Proposed conceptual framework in which orthobiologic response is interpreted through the interaction among seed properties, systemic host biology, and local tissue receptivity.

Knee OA was used as the primary disease context for applying the framework because it combines substantial biological heterogeneity, whole-joint involvement, multiple pain mechanisms, validated clinical and imaging outcomes, and an expanding literature on platelet-rich plasma (PRP), marrow-derived products, adipose-derived therapies, biomarkers, and precision-medicine approaches (Tang et al., 2025; Navani et al., 2025). The proposed interaction among seed properties, systemic host biology, and local tissue receptivity is illustrated in Figure 1.

### 3.2 Why product characterization alone may not explain response heterogeneity

Three recurring observations in the reviewed literature support extending product characterization to include host- and tissue-level determinants of therapeutic response. First, the seed itself was highly heterogeneous. PRP could not be regarded as a uniform intervention because preparations differed in platelet concentration, leukocyte content, erythrocyte contamination, activation method, processing technique, dose, injection volume, and administration schedule (Andia et al., 2025). Likewise, marrow-derived products differed according to harvest site, aspiration technique, aspirated volume, peripheral blood contamination, processing method, concentration, and cellular composition (Park et al., 2025; Maruccia et al., 2026). Adipose-derived stromal cells, stromal vascular fraction, and microfragmented adipose tissue also represented biologically distinct interventions rather than interchangeable forms of a single therapeutic category (Batista et al., 2026).

Second, the biological effects attributed to orthobiologics appeared to be context-dependent rather than autonomous. The concept of mesenchymal stromal cells as an “injury drugstore” shifted their interpretation from passive structural building blocks toward dynamic paracrine, trophic, immunomodulatory, and homeostatic mediators whose activity may depend on the biological environment into which they are delivered (Caplan and Correa, 2011). Under this model, treatment activity may be influenced by inflammatory signaling, metabolic status, tissue damage, mechanical loading, vascular conditions, and interactions with resident cells.

Third, heterogeneous clinical outcomes were observed across orthobiologic trials and evidence syntheses despite the use of broadly similar nominal product categories. Orthobiologic trials and evidence syntheses in knee OA demonstrated a combination of clinically meaningful symptomatic improvement, neutral between-group findings, substantial improvement in comparator groups, variable structural outcomes, and differences related to product processing and trial architecture (Batista et al., 2026; Cao et al., 2025; Yin et al., 2025; Mautner et al., 2023). These findings were difficult to reconcile using a seed-only model.

### 3.3 Seed: from a nominal label to a functional biological entity

The reviewed literature supports detailed functional characterization of the seed rather than reliance on nominal product labels alone. Terms such as PRP, bone marrow aspirate concentrate, “stem cells,” or “adipose therapy” were insufficient to characterize the biological intervention unless accompanied by information on tissue source, harvest conditions, processing pathway, cellular and molecular composition, dose, viability, biological potency, administration schedule, and delivery technique (Andia et al., 2025; Navani et al., 2025).

This distinction was particularly important for marrow-derived products. Bone marrow aspirate should be considered a biological product in its own right and not merely a precursor to bone marrow aspirate concentrate. The biological quality of the marrow-derived seed begins during aspiration, and differences in aspiration technique, harvest site, aspirated volume, peripheral blood contamination, and subsequent processing may alter cellular yield and product composition (Park et al., 2025; Maruccia et al., 2026).

A similar principle applied to PRP. The PRP literature increasingly describes a research ecosystem composed of multiple preparations rather than a single standardized intervention. This variability limits direct comparisons across trials and supports the need for precise reporting of platelet and leukocyte concentrations, erythrocyte content, activation procedures, injected volume, number of injections, and relevant recipient characteristics (Andia et al., 2025).

Product heterogeneity was even more pronounced among adipose-derived interventions. The systematic review by Batista et al. synthesized 19 randomized controlled trials and analyzed adipose-derived stromal cells, stromal vascular fraction, and microfragmented adipose tissue as distinct biological categories. The review identified meaningful clinical signals for pain, function, and safety, particularly in studies of adipose-derived stromal cells, but also documented major heterogeneity in preparation methods, dose, comparator, outcome assessment, and follow-up (Batista et al., 2026).

Individual randomized trials of autologous and allogeneic adipose-derived stromal-cell preparations further illustrated this variability. These studies differed in cell source, expansion process, dose, placebo or active comparator, participant characteristics, OA severity, and follow-up duration, reinforcing that even products within the same broad cellular category should not automatically be considered biologically equivalent (Lee et al., 2019; Freitag et al., 2024; Pers et al., 2025).

### 3.4 Systemic soil: host biological readiness before intervention

The reviewed literature characterizes patients with knee OA as having heterogeneous systemic metabolic, inflammatory, behavioral, and clinical profiles that may be relevant to biological responsiveness. These profiles reflect a pre-existing systemic environment shaped by metabolic health, systemic inflammation, sleep, medication exposure, nutrition, physical conditioning, psychological state, health behavior, and comorbid disease.

Within the proposed framework, metabolic-inflammatory status was therefore considered a candidate systemic-soil domain. Contemporary models recognize OA as a heterogeneous disease in which metabolic and inflammatory mechanisms interact with biomechanical and structural processes (Tang et al., 2025). Obesity, insulin resistance, altered body composition, and chronic low-grade inflammation may therefore influence both the characteristics of autologous biological products and the responsiveness of the recipient environment (Lana et al., 2024; Navani et al., 2025).

The SDIMMMER model provided a structured host-oriented approach incorporating sleep, diet, interactions with the microbiome, metabolism, medications, laboratory assessment, mental health, exercise, and rehabilitation (Lana et al., 2024). Although this complete framework has not yet been prospectively validated as a therapeutic package in randomized orthobiologic trials, it highlights the possibility that regenerative response may be modified by the broader physiological condition of the patient.

Sleep and chronobiology were consequently retained as potentially relevant systemic-soil domains because of their relationships with neuroimmune regulation, pain processing, recovery, and health behavior. However, evidence directly demonstrating that sleep optimization improves the efficacy of a specific orthobiologic intervention remains insufficient (Lana et al., 2024).

Selected microbiome-related mechanisms may also belong within systemic soil. Evidence concerning the gut–joint axis suggests biologically plausible interactions among intestinal dysbiosis, barrier dysfunction, systemic immune tone, and musculoskeletal inflammation. Nevertheless, this remains a predominantly translational domain, with insufficient evidence to support routine microbiome-directed intervention as a validated component of orthobiologic treatment (Longo et al., 2024).

The functional and rehabilitative context was also incorporated within systemic soil. Regenerative treatments are administered to patients who remain mechanically active and differ substantially in muscle strength, physical capacity, movement behavior, adherence, and ability to participate in rehabilitation. Rehabilitation therefore represents not only a post-procedure intervention but also part of the biological and mechanical environment in which treatment effects develop (Lana et al., 2024; Navani et al., 2025).

### 3.5 Local tissue soil: structural, inflammatory, sensory, and mechanical receptivity

Local tissue soil was defined as the biological condition of the joint receiving the intervention. In knee OA, the target tissue could not be interpreted through a cartilage-only model. OA involves interactions among cartilage, synovium, joint capsule, subchondral bone, menisci, ligaments, periarticular muscles, sensory structures, and local adipose tissues (Tang et al., 2025; Harkey et al., 2026).

The synovium and joint capsule were incorporated as relevant components of local tissue soil based on evidence linking synovitis with inflammatory signaling, symptoms, structural progression, and nociceptive activation. Synovitis contributes to inflammatory signaling, symptom expression, structural progression, and activation of nociceptive pathways. A joint characterized by persistent synovitis and inflammatory-sensory activation may therefore represent a biologically different target from a joint with similar radiographic severity but limited synovial activity (Panichi et al., 2024).

The subchondral compartment also represented an important biological and sensory domain. Experimental evidence demonstrated that abnormal subchondral bone remodeling may promote sensory innervation and OA-related pain through osteoclast-mediated signaling. This finding suggests that subchondral abnormalities may contribute not only to structural progression but also to local sensory amplification (Zhu et al., 2019).

Pain phenotype was treated as a cross-cutting modifier rather than as a strictly local tissue characteristic because it reflects interactions among peripheral nociceptive input, local joint pathology, central nervous system processing, and psychosocial factors. Neuropathic-like symptoms, catastrophizing, and central sensitization-related signs have been associated with greater pain burden and functional impairment in knee OA while often showing limited correspondence with conventional radiographic severity (Salaffi et al., 2025). Accordingly, pain phenotype may influence the clinical expression of local tissue pathology and the interpretation of treatment response without being reducible to the local joint environment itself.

A similar boundary applies to functional variables: periarticular muscle structure and function were considered predominantly local mechanical characteristics, whereas rehabilitation capacity, adherence, and broader physical conditioning were treated as cross-cutting functional or host-level factors.

Imaging represented the most clinically accessible current approach for characterizing local tissue soil. Contemporary imaging research increasingly extends beyond cartilage thickness and joint-space narrowing to include synovitis, effusion, bone marrow lesions, meniscal damage or extrusion, subchondral changes, infrapatellar fat-pad abnormalities, and periarticular muscle characteristics (Herrera et al., 2024; Harkey et al., 2026). Selected MRI findings may provide prognostic or predictive information, although their role in selecting patients for specific orthobiologic interventions remains incompletely validated.

### 3.6 Biomarkers and molecular endotypes

Candidate biomarkers of local tissue soil included synovial inflammatory mediators, matrix-turnover markers, imaging-derived features, and exploratory neuro-sensory indicators. Synovial-fluid studies identified potential roles for interleukin-6, interleukin-8, tumor necrosis factor-α, vascular endothelial growth factor, matrix metalloproteinase-3, tissue inhibitor of metalloproteinases-1, leptin, cartilage oligomeric matrix protein, and chondroitin sulfate-related markers (Boffa et al., 2021; Hannani et al., 2024). Candidate biochemical, imaging, and neuro-sensory markers of local tissue soil are summarized in Table 1.

**TABLE 1:** Candidate biomarkers for local tissue soil in knee osteoarthritis.

Biomarker/marker domain	Specimen or modality	Biological meaning	Current maturity	Proposed role in the framework
IL-6 (Boffa et al., 2021; Hannani et al., 2024)	Synovial fluid	Inflammatory activation/synovial hostility	Higher-priority translational candidate	Candidate marker of inflammatory local soil
IL-8 (Boffa et al., 2021; Hannani et al., 2024)	Synovial fluid	Chemotactic inflammatory signaling	Higher-priority translational candidate	Candidate marker of inflammatory local soil
TNF-α (Boffa et al., 2021; Hannani et al., 2024)	Synovial fluid	Pro-inflammatory catabolic signaling	Higher-priority translational candidate	Candidate marker of inflammatory local soil
VEGF (Boffa et al., 2021; Hannani et al., 2024)	Synovial fluid	Vascular/inflammatory remodeling	Higher-priority translational candidate	Candidate marker of activated synovial-local environment
MMP-3 (Boffa et al., 2021; Hannani et al., 2024)	Synovial fluid	Matrix degradation/catabolic activity	Higher-priority translational candidate	Candidate marker of hostile matrix turnover
TIMP-1 (Boffa et al., 2021; Hannani et al., 2024)	Synovial fluid	Matrix remodeling balance	Higher-priority translational candidate	Candidate marker of remodeling state
Leptin (Boffa et al., 2021; Hannani et al., 2024)	Synovial fluid	Adipokine-related inflammatory signaling	Higher-priority translational candidate	Candidate marker linking metabolic and local tissue biology
COMP (Boffa et al., 2021; Hannani et al., 2024)	Synovial fluid/serum	Cartilage matrix turnover	Supportive candidate	Candidate marker of cartilage-related local tissue burden
C4S (Boffa et al., 2021; Hannani et al., 2024)	Synovial fluid	Cartilage/matrix-related burden	Supportive candidate	Candidate marker of matrix-related local tissue status
Effusion-synovitis (Herrera et al., 2024; Harkey et al., 2026)	MRI	Synovial inflammatory burden	Higher-priority imaging biomarker	Candidate imaging marker of inflammatory local soil
Hoffa-synovitis (Herrera et al., 2024; Harkey et al., 2026)	MRI	Inflammatory activity in infrapatellar fat pad/joint microenvironment	Higher-priority imaging biomarker	Candidate imaging marker of activated local tissue soil
Bone marrow lesions (Herrera et al., 2024; Harkey et al., 2026)	MRI	Subchondral bone stress/remodeling/pain-related biology	Higher-priority imaging biomarker	Candidate imaging marker of subchondral local soil
Meniscal damage/extrusion (Herrera et al., 2024; Harkey et al., 2026)	MRI	Structural–mechanical dysfunction of local tissue environment	Higher-priority imaging biomarker	Candidate imaging marker of mechanically hostile local soil
NGF (Zhu et al., 2019; Boffa et al., 2021)	Synovial fluid/tissue/exploratory	Neuroimmune pain signaling	Exploratory/emerging	Candidate marker of neuro-sensory local soil
CGRP (Boffa et al., 2021)	Synovial fluid/tissue/exploratory	Sensory nerve activation/neurogenic inflammation	Exploratory/emerging	Candidate marker of capsular–sensory local soil
Substance P (Boffa et al., 2021)	Synovial fluid/tissue/exploratory	Neurogenic pain and inflammatory amplification	Exploratory/emerging	Candidate marker of neuropathic-like local pain biology

The maturity categories used in this table describe the relative translational and operational maturity of individual biomarkers and should not be interpreted as equivalent to the evidence-status classification applied to framework-level propositions in Supplementary Table S3.

No single biomarker or routine-ready panel was sufficiently validated to characterize regenerative readiness or reliably predict orthobiologic response. Current biomarkers were therefore better interpreted as research, stratification, and trial-enrichment tools than as established clinical tests (Boffa et al., 2021; Hannani et al., 2024).

The progression from isolated biochemical markers toward molecular endotypes offered a more advanced model for future patient classification. Genomic, epigenomic, proteomic, and metabolomic findings suggest that patients sharing the same clinical diagnosis may have different underlying biological architectures (Hannani et al., 2024). This concept is compatible with the Seed–Systemic Soil–Local Tissue Soil framework because it supports the transition from broad OA phenotypes toward biologically defined therapeutic subgroups.

The emerging concept of pre-osteoarthritis extends this reasoning to earlier disease stages. Biological vulnerability may exist before advanced radiographic disease or irreversible whole-joint disruption, raising the possibility that regenerative interventions could produce more consistent effects when applied to a biologically vulnerable but still receptive tissue environment (del Río, 2025).

**FIGURE 2:**
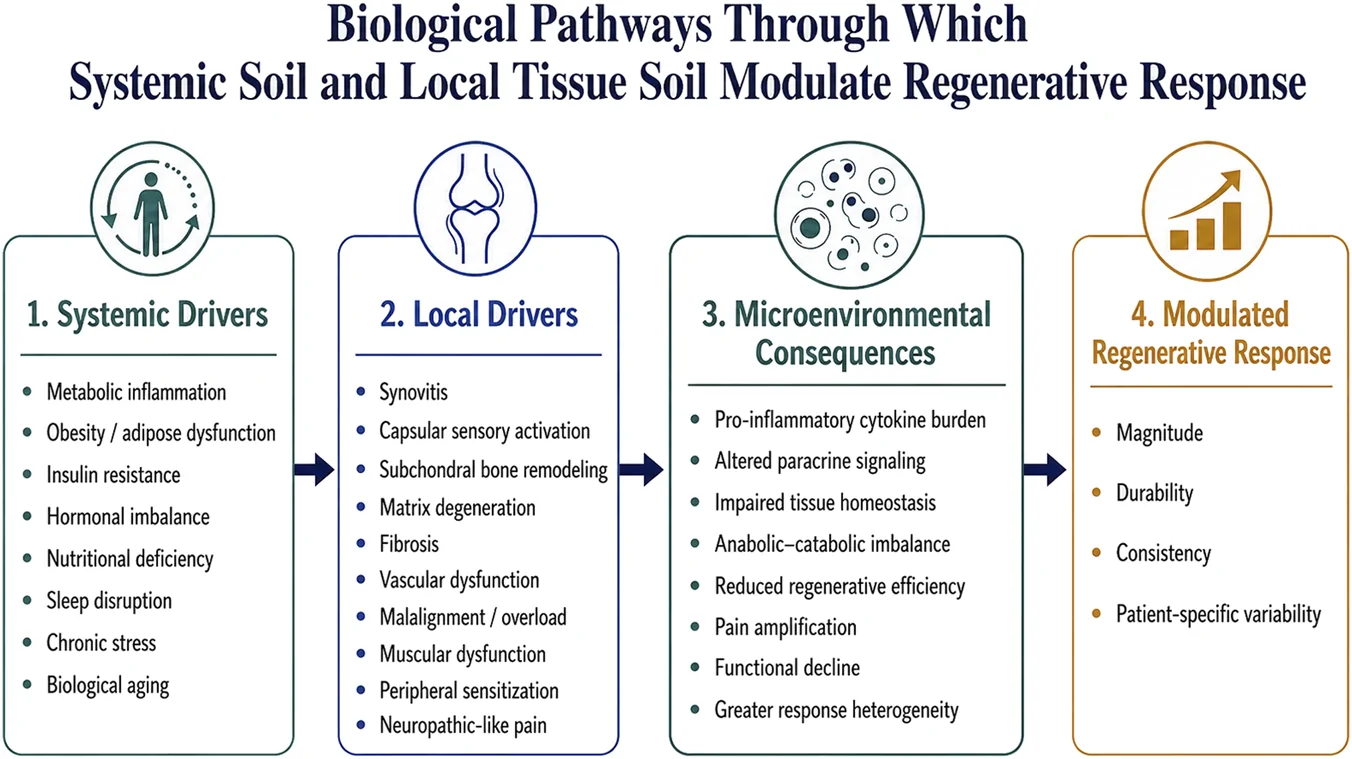
Biological pathways through which systemic soil and local tissue soil modulate regenerative response. Systemic and local biologic drivers may alter the regenerative microenvironment and influence response magnitude, durability, and consistency. The figure is intended as a translational schema rather than as a validated causal algorithm.

The proposed pathways through which systemic and local drivers may alter inflammatory signaling, paracrine communication, tissue homeostasis, pain amplification, and the magnitude or durability of regenerative response are summarized in Figure 2. This figure should be interpreted as a translational schema rather than as a prospectively validated causal model.

### 3.7 Knee osteoarthritis as the primary evidence context

Knee OA provides a particularly informative clinical context for applying the proposed framework because it combines high prevalence, clinical heterogeneity, multiple structural and pain phenotypes, diverse orthobiologic interventions, and a broad range of validated outcomes (Tang et al., 2025; Batista et al., 2026).

The adipose-derived literature illustrated both the potential and the limitations of current regenerative interventions. The systematic review by Batista et al. identified meaningful signals for pain reduction, functional improvement, and safety across 19 randomized controlled trials, particularly among studies of adipose-derived stromal cells. However, it also found substantial methodological and biological heterogeneity and no uniformly demonstrated structural regeneration of articular cartilage (Batista et al., 2026).

The broader mesenchymal stromal-cell literature similarly suggested symptomatic benefit in at least part of the available evidence while confirming variability in cell source, dose, processing, comparator, outcome selection, follow-up duration, and methodological quality (Cao et al., 2025). These findings support the existence of clinical activity but do not establish a universal or consistently regenerative effect across products and patients.

Knee OA also demonstrated why diagnosis alone is insufficient for precision treatment. Within the same diagnostic category, one patient may present with obesity, metabolic dysfunction, sleep disturbance, active synovitis, bone marrow lesions, neuropathic-like pain, central sensitization, muscle weakness, and a poorly characterized biological product. Another patient may have a more favorable metabolic profile, predominantly peripheral nociceptive pain, limited inflammatory activity, preserved tissue architecture, and a more standardized intervention. These patients should not necessarily be expected to respond identically despite sharing the same diagnostic label.

### 3.8 Clinical signal, contextual effects, and active comparators

A recurring interpretive theme across the reviewed clinical literature was the coexistence of symptomatic clinical signals and uncertainty regarding the magnitude of the product-specific treatment effect. Meta-analyses of randomized trials have reported improvements in pain and function after mesenchymal stromal-cell therapy while simultaneously demonstrating variability in effect size, certainty, and methodological quality (Cao et al., 2025).

Contextual effects represented an important source of symptomatic improvement. A systematic review and meta-analysis focusing specifically on contextual effects concluded that a substantial proportion of the clinical change observed following mesenchymal stromal-cell injections may be attributable to placebo-related and nonspecific procedural effects, while the incremental product-specific contribution may be more modest (Yin et al., 2025).

Active comparators further complicated interpretation. A phase 3 randomized trial found that cell-based injections were not superior to corticosteroid injection for knee OA pain at 1 year, demonstrating that a biologically complex intervention does not necessarily produce greater average clinical benefit than an established active comparator (Mautner et al., 2023).

Hyaluronic acid and PRP should likewise not be regarded as inert control interventions. Umbrella and network meta-analyses indicated that hyaluronic acid, PRP, BMAC, and combination strategies may produce clinically relevant improvements in pain and function, particularly over intermediate follow-up periods (Bruyère et al., 2025; Qiao et al., 2023; Jawanda et al., 2024). Consequently, failure to demonstrate superiority over an active comparator does not necessarily indicate biological inactivity, just as large within-group improvements do not establish product-specific regeneration.

These findings support the need to distinguish among total clinical improvement, contextual improvement, comparator-related improvement, and the incremental effect attributable specifically to the biological product.

### 3.9 Proposed framework for future operationalization

To improve the testability, feasibility, and prospective validation of the framework, candidate variables were organized into near-term core domains and exploratory domains. Core domains were defined as variables that are clinically measurable, reasonably reproducible, and supported by sufficient human evidence to justify prospective stratification. Five core domains were prioritized: (1) rigorous seed characterization, including source, processing, composition, dose, viability, and delivery characteristics; (2) systemic metabolic-inflammatory status; (3) local structural and inflammatory joint status, including disease severity and synovitis; (4) pain phenotype as a cross-cutting clinical modifier; and (5) functional and rehabilitative status.

Exploratory domains included microbiome-related factors, chronobiology, multi-omic signatures, molecular endotypes, and emerging neuro-sensory biomarkers. These variables may have biological relevance but currently lack sufficient evidence or operational maturity for routine incorporation into initial prospective validation studies. Sleep may be assessed as a clinically relevant host characteristic because of its associations with pain, recovery, and neuroimmune regulation; however, its role as a modifier of orthobiologic efficacy remains unproven and it should not be interpreted as a prerequisite for treatment.

The core domains were mapped across the Seed–Systemic Soil–Local Tissue Soil framework while preserving their conceptual boundaries. Seed characterization represents the technical and biological definition of the intervention. Metabolic-inflammatory status primarily characterizes systemic host biology, whereas structural severity and synovitis characterize local tissue conditions. Pain phenotype was treated as a cross-cutting modifier because it reflects interactions among peripheral joint pathology, nociceptive processing, central nervous system mechanisms, and psychosocial factors rather than a strictly local tissue characteristic. Functional status and rehabilitation similarly intersect host capacity, local biomechanics, and the therapeutic context.

Within this framework, regenerative readiness should be interpreted as a multidimensional research construct rather than as a binary clinical state. Different combinations of product characteristics, systemic host factors, local joint conditions, pain mechanisms, and functional capacity may coexist within the same diagnostic category. Importantly, the presence of a potentially unfavorable factor does not establish that correcting that factor will improve orthobiologic efficacy, nor does its absence define a validated state of regenerative readiness.

Accordingly, the proposed model favors prospective multimodal characterization and prespecified stratification rather than reliance on a single biomarker, imaging feature, or host characteristic. Initial validation studies may benefit from focusing on a limited number of core variables rather than attempting to assess the entire framework simultaneously. The principal components of the Seed–Systemic Soil–Local Tissue Soil framework, together with their biological relevance and potential management considerations when independently clinically indicated, are summarized in Table 2.

**TABLE 2:** Candidate components of the seed–systemic soil–local tissue soil framework in knee osteoarthritis.

Domain	Component	Biological relevance	Clinical relevance	Potential management considerations when independently clinically indicated
Systemic soil	Low-grade systemic inflammation (Tang et al., 2025; Lana et al., 2024)	Alters immune tone and repair signaling	May reduce responsiveness and increase pain persistence	Metabolic optimization; anti-inflammatory lifestyle strategies
Systemic soil	Obesity/body composition (Tang et al., 2025; Lana et al., 2024)	Influences inflammatory burden, biomechanics, and adipokine signaling	Associated with worse outcomes and altered load behavior	Weight reduction; body composition improvement
Systemic soil	Insulin resistance/metabolic dysfunction (Tang et al., 2025; Lana et al., 2024)	Impairs tissue homeostasis and regenerative efficiency	May contribute to lower consistency of response	Glycemic/metabolic control
Systemic soil	Sleep/chronobiology (Lana et al., 2024)	Influences pain processing, recovery, inflammation, and neuroimmune balance	Associated with worse pain and function	Sleep assessment and targeted sleep improvement
Systemic soil	Nutrition (Lana et al., 2024)	Shapes metabolic health and tissue recovery capacity	May influence biologic readiness	Nutritional optimization
Systemic soil	Comorbidities (Lana et al., 2024)	Affect host resilience and systemic biologic balance	May modify indication, risk, and expected response	Clinical stabilization of comorbidities
Local tissue soil	Synovitis (Panichi et al., 2024)	Reflects inflammatory activation within the target tissue	Strongly linked to pain and tissue hostility	Phenotype-guided local anti-inflammatory strategies
Local tissue soil	Subchondral bone remodeling (Zhu et al., 2019)	Contributes to pain and altered joint biology	May influence progression and responsiveness	Offloading and phenotype-specific intervention
Local tissue soil	Muscular dysfunction (Tang et al., 2025; Harkey et al., 2026)	Alters load distribution and joint control	Contributes to pain and reduced function	Strengthening and motor control rehabilitation
Local tissue soil	Mechanical overload/malalignment (Tang et al., 2025)	Maintains a hostile tissue environment	Can undermine durability of response	Load modulation and biomechanical correction
Local tissue soil	Pain phenotype/sensitization (Salaffi et al., 2025)	Reflects sensory dysregulation and pain amplification	May predict poorer symptom–structure coupling	Multimodal pain-directed management
Seed	PRP (Andia et al., 2025)	Blood-derived signaling product with heterogeneous composition	Frequently used but variably characterized	Standardize preparation and document composition
Seed	BMA (Maruccia et al., 2026)	Raw marrow-derived biologic substrate	Harvest quality may affect therapeutic potential	Optimize aspiration technique and site
Seed	BMAC (Park et al., 2025)	Concentrated marrow-derived product	May provide enriched biologic content but remains heterogeneous	Standardize harvest and processing
Seed	Adipose-derived products (Batista et al., 2026)	Includes ADSCs, SVF, MFAT and related classes	Distinct biologic categories with different profiles	Separate categories explicitly and report source/processing
Seed	Dose and schedule (Navani et al., 2025; Andia et al., 2025)	Modulate biologic exposure and potential effect	May alter magnitude and durability of response	Rational dose/schedule matching

Potential management considerations reflect general clinical or biological rationale and should be considered only when independently clinically indicated. They should not be interpreted as evidence that modifying the corresponding factor improves orthobiologic efficacy. The evidence status of the principal framework propositions is summarized in Supplementary Table S3.

**FIGURE 3:**
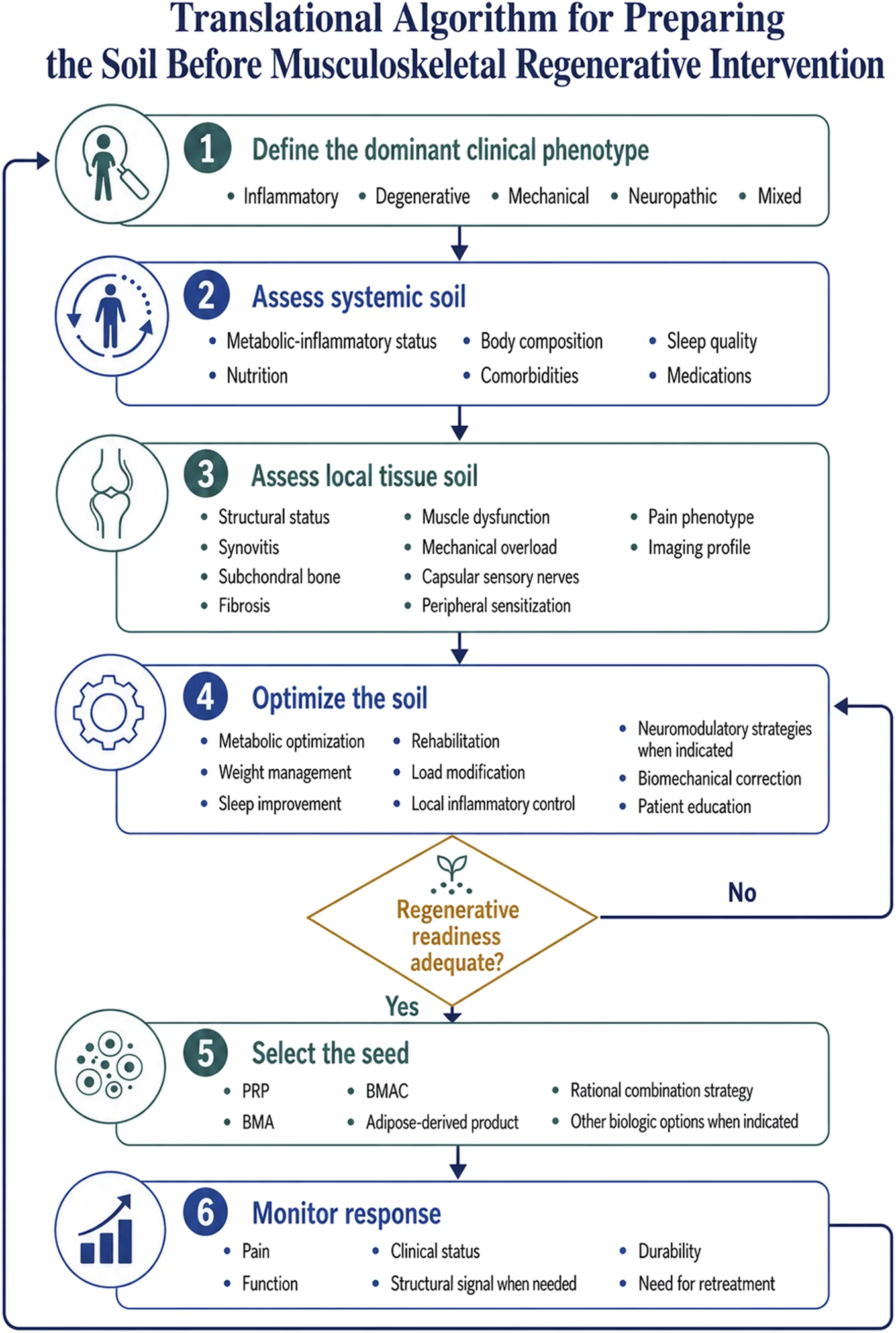
Hypothesis-generating research workflow for prospective evaluation of the Seed–Systemic Soil–Local Tissue Soil framework in knee osteoarthritis. The proposed workflow integrates phenotype definition, systemic and local tissue assessment, characterization of potentially modifiable factors, standardized seed characterization, and longitudinal response monitoring. Management of modifiable factors is considered only when independently clinically indicated. The figure represents a research framework and not a validated clinical treatment algorithm.

A hypothesis-generating research workflow integrating clinical phenotype definition, systemic and local tissue assessment, characterization of potentially modifiable factors, standardized seed characterization, documentation of rehabilitation and other co-interventions, and longitudinal response monitoring is presented in Figure 3. This workflow is intended to support prospective testing of treatment-by-phenotype and treatment-by-biomarker interactions and should not be interpreted as a validated clinical treatment algorithm.

## 4 Discussion

This integrative review proposes that heterogeneity in orthobiologic treatment response in knee OA may be interpreted through the interaction among product characteristics, systemic host biology, and local tissue receptivity. The principal contribution is not a claim that systemic or local soil optimization has already been definitively established as an effective therapeutic doctrine. Rather, our synthesis suggests that product characterization, although indispensable, may not by itself account for the heterogeneity observed across orthobiologic trials and clinical practice.

The conventional question of “what should be injected?” was essential during the early development of orthobiologics. It enabled investigators to establish initial safety, biological plausibility, and clinical signals for PRP, marrow-derived products, mesenchymal stromal cells, and adipose-derived therapies. However, continued reliance on a predominantly product-centered framework may conceal the fact that the intervention, the recipient, and the target tissue are all biologically variable.

### 4.1 The seed remains essential but requires improved characterization

The proposed framework does not reduce the importance of product quality. On the contrary, it requires a more rigorous and functionally meaningful definition of the seed. PRP, BMA, BMAC, adipose-derived stromal cells, stromal vascular fraction, and microfragmented adipose tissue should not be interpreted as homogeneous or interchangeable interventions.

In PRP research, inconsistent preparation and reporting remain major barriers to precision interpretation. Variation in platelet concentration, leukocyte and erythrocyte content, activation method, injected volume, dosing schedule, and participant selection may produce preparations with different biological and clinical properties (Andia et al., 2025).

Similar challenges occur in marrow-derived therapies, in which the aspiration process itself may influence the composition and biological quality of the final product. The distinction between BMA and BMAC must therefore be preserved, and studies should report aspiration method, harvest site, aspirated volume, processing strategy, and final product characteristics (Park et al., 2025; Maruccia et al., 2026).

Adipose-derived therapies further illustrate the importance of maintaining biological distinctions. Expanded adipose-derived stromal cells, non-expanded stromal vascular fraction, and mechanically processed adipose tissue differ in processing, cellular composition, regulatory status, mechanisms, and potential biological fitness. Combining these interventions under a generic label such as “adipose stem-cell therapy” may obscure clinically relevant differences (Batista et al., 2026).

The concept of the mesenchymal stromal cell as an injury-responsive signaling system also suggests that product characterization should move beyond cell number alone. Viability, secretory capacity, immunomodulatory function, senescence, potency, and responsiveness to the recipient environment may all influence therapeutic activity (Caplan and Correa, 2011). Future studies should therefore consider functional potency measures when feasible rather than relying exclusively on nominal cell identity or total cellular dose.

### 4.2 Systemic soil should be treated as a potential modifier rather than a proven prerequisite

The systemic-soil component reflects the biological condition of the recipient before intervention. This proposal is supported by the recognition that metabolism, inflammation, sleep, medications, nutrition, physical capacity, and comorbidities may influence tissue homeostasis, immune regulation, and recovery.

However, the distinction between biological plausibility and clinical proof is essential. Host-oriented frameworks such as SDIMMMER provide a useful conceptual structure, but they do not yet demonstrate that correcting every systemic domain before an orthobiologic procedure improves treatment efficacy (Lana et al., 2024). Current evidence therefore supports systemic factors as candidate moderators, mediators, prognostic variables, or stratification domains—not as mandatory prerequisites with established causal effects. Accordingly, the present framework should be viewed as complementary to, rather than a replacement for, SDIMMMER: it incorporates host-oriented factors within a broader explanatory structure that also distinguishes seed characteristics and local tissue receptivity.

This distinction has practical consequences. General health optimization may be reasonable as part of comprehensive OA management, but claims that sleep improvement, microbiome manipulation, metabolic correction, or laboratory-guided optimization will necessarily enhance the efficacy of an injected product would exceed the current evidence.

The most informative next step may not be to test the entire systemic-soil concept as a bundled intervention. Prospective studies should initially investigate individual, measurable domains such as metabolic-inflammatory status, obesity, glycemic dysfunction, sleep disturbance, medication exposure, and physical conditioning. Prespecified interaction analyses could then determine whether these variables modify response to a standardized seed.

### 4.3 Local tissue soil may explain why the same diagnosis produces different responses

Knee OA is particularly suitable for the framework because the clinical diagnosis encompasses multiple structural and biological states. Similar radiographic grades may coexist with different levels of synovitis, subchondral remodeling, meniscal pathology, mechanical overload, muscle dysfunction, pain sensitization, and metabolic disease.

A biological product delivered into a joint with persistent synovitis, extensive bone marrow lesions, severe malalignment, substantial meniscal extrusion, and sensory amplification encounters a different environment from the same product delivered into a joint with preserved alignment, limited inflammatory activity, and less advanced tissue disruption.

Synovial inflammation is particularly relevant because many proposed orthobiologic mechanisms are immunomodulatory rather than directly regenerative. If a product acts partly through modulation of inflammatory signaling, baseline synovial phenotype may influence both the magnitude and duration of response (Panichi et al., 2024).

Subchondral bone may also determine therapeutic receptivity. Abnormal remodeling and sensory innervation provide a plausible explanation for persistent pain despite treatments directed primarily toward the intra-articular compartment (Zhu et al., 2019). An intra-articular product may have limited ability to reverse pain generated predominantly by advanced subchondral or mechanically maintained pathology.

Pain phenotype adds another layer of complexity. Neuropathic-like symptoms, catastrophizing, and central sensitization may amplify symptoms independently of peripheral structural severity (Salaffi et al., 2025). A patient with predominantly centrally amplified pain may report limited improvement after an intervention whose main effects are local and anti-inflammatory, even when that intervention produces measurable intra-articular biological activity.

This may explain why structural, inflammatory, and symptomatic outcomes frequently diverge. Symptomatic improvement does not necessarily indicate cartilage regeneration, and absence of major pain relief does not necessarily indicate complete biological inactivity. Trials should therefore distinguish clearly among pain modulation, functional improvement, inflammatory change, structural modification, and tissue regeneration.

### 4.4 Imaging and biomarkers may convert the soil metaphor into a testable model

For the proposed framework to progress beyond metaphor, systemic and local soil could become at least partly measurable. Imaging currently offers the most practical approach to local tissue characterization.

MRI permits assessment of cartilage, synovitis, effusion, bone marrow lesions, meniscal pathology, subchondral bone, infrapatellar fat pad, and periarticular muscle. However, the predictive role of individual imaging features for orthobiologic response remains uncertain (Herrera et al., 2024; Harkey et al., 2026). Most studies have used imaging to describe baseline structure or investigate disease progression rather than prospectively test treatment-effect modification.

Biochemical markers may complement imaging by capturing inflammatory, metabolic, matrix-related, and neuro-sensory processes. Synovial-fluid biomarkers provide biologically relevant information about the local joint environment, but variability in sample collection, laboratory methods, disease stage, and outcome definitions currently limits routine implementation (Boffa et al., 2021).

The progression toward molecular endotypes may eventually allow identification of biologically distinct OA subgroups that respond differently to specific seeds (Hannani et al., 2024). Nevertheless, endotype-based treatment selection remains an emerging research objective rather than an established clinical pathway.

A realistic near-term strategy would combine a limited number of reproducible variables, including metabolic status, sleep burden, standardized pain phenotype, radiographic and MRI characteristics, synovitis assessment, physical function, and rigorous product characterization. Such an approach could provide a feasible foundation for prospective stratification without requiring an impractically large multi-omic panel.

### 4.5 Clinical improvement must be separated from product-specific effects

One of the most important implications of this synthesis concerns interpretation of efficacy. Intra-articular interventions are associated with contextual effects involving expectations, clinical interaction, needle-based procedures, natural symptom fluctuation, regression toward the mean, and post-procedure behavior.

The analysis by Yin et al. indicated that contextual effects may account for a substantial proportion of symptomatic improvement observed in mesenchymal stromal-cell trials (Yin et al., 2025). This does not establish that the biological products are inactive. Instead, it demonstrates that the total observed response is composed of multiple elements and that product-specific efficacy could be estimated through appropriately controlled comparisons.

Active comparators also require careful interpretation. Corticosteroids, hyaluronic acid, PRP, and BMAC may all improve pain or function under certain conditions (Mautner et al., 2023; Qiao et al., 2023; Jawanda et al., 2024; Bruyère et al., 2025). Therefore, a trial showing no superiority over an active comparator should not be interpreted in the same way as a trial showing no difference from a credible inert placebo.

The phase 3 trial comparing cell-based treatments with corticosteroid injection illustrates this issue. The absence of superiority at 1 year challenges assumptions that greater biological complexity necessarily produces greater average clinical benefit (Mautner et al., 2023). At the same time, this finding does not establish equivalence across every product, phenotype, disease stage, or tissue environment.

The seed–soil framework offers a possible explanation for such findings. A clinically active seed may demonstrate only modest average superiority when evaluated across a biologically heterogeneous population. If a treatment produces meaningful benefit primarily in a specific systemic or local tissue soil, conventional group-average analyses may dilute that effect.

### 4.6 Extending product trials through biological stratification

A potential translational application of the framework is to extend conventional product-focused trials through prespecified biological stratification. In addition to asking whether a clearly characterized intervention improves an average outcome, future studies could test whether its effects differ across predefined systemic and local tissue environments.

The conceptual extension from a conventional product-focused perspective to an integrated Seed–Systemic Soil–Local Tissue Soil perspective is summarized in Table 3. In this integrated perspective, diagnosis remains an important starting point but is complemented by phenotype and biological context; product characterization remains essential and is considered alongside host and local tissue biology; pain phenotype becomes part of the biological terrain; and rehabilitation is incorporated into the broader therapeutic context.

**TABLE 3:** Extending product-centered logic through an integrated seed–systemic soil–local tissue soil framework.

Conventional product-focused perspective	Integrated seed–soil perspective
What are we injecting?	Into what biological terrain are we intervening?
Diagnosis is the main treatment guide	Diagnosis is only the starting point; phenotype and biologic context matter
Product characterization is central	Product characterization remains essential and is integrated with host and local tissue biology
Failure implies inadequate product choice	Failure may reflect mismatch between seed and soil
Structural imaging dominates interpretation	Structural, inflammatory, sensory, and functional tissue biology are all relevant
Pain is treated mainly as a symptom outcome	Pain phenotype is part of biologic terrain and may modify response
Rehabilitation is adjunctive aftercare	Rehabilitation is part of the therapeutic terrain
One product may be broadly applied across similar diagnoses	Product effects may be evaluated across predefined host and tissue characteristics
Trials seek average efficacy across heterogeneous patients	Trials may incorporate prespecified biological stratification

Biological stratification studies would require careful design. First, the product would need to be standardized and reported with sufficient technical detail. Second, a limited number of prespecified soil variables could be measured before treatment. Third, pain mechanisms and contextual factors could be incorporated into the analytical framework. Fourth, rehabilitation and other co-interventions could be standardized or explicitly documented. Finally, prospective statistical analyses could test treatment-by-phenotype or treatment-by-biomarker interactions.

Adaptive, enrichment, factorial, or biomarker-stratified designs may be particularly useful. A trial could, for example, investigate a standardized cellular product in patients stratified by metabolic-inflammatory status and MRI-detected synovitis. Another could evaluate whether neuropathic-like pain features modify the symptomatic response to an otherwise biologically active intra-articular intervention.

The strongest immediate studies may be those that test one layer of the framework under disciplined conditions rather than attempting to validate every component simultaneously. Testing the complete Seed–Systemic Soil–Local Tissue Soil model as a single therapeutic package would require very large samples and could obscure which individual factors are causally important.

### 4.7 Clinical implications

The framework has several potential clinical implications, although it should not yet be interpreted as a validated treatment algorithm. First, it highlights that products within a broad orthobiologic category should not automatically be assumed to be biologically equivalent. Clinical interpretation may therefore benefit from transparent characterization of processing, dose, composition, and relevant regulatory characteristics.

Second, the framework emphasizes assessment of the patient beyond the diagnostic label alone. Metabolic health, sleep, physical capacity, medication exposure, pain phenotype, synovitis, mechanical alignment, structural disease, and rehabilitation feasibility may influence treatment expectations and interpretation of outcomes.

The model also supports more realistic communication with patients. Orthobiologic treatment may improve pain or function without regenerating cartilage. Failure to demonstrate cartilage regeneration does not necessarily eliminate symptomatic value, but symptomatic improvement should not be presented as evidence of tissue restoration.

Finally, the framework reinforces that orthobiologics should generally be integrated into comprehensive OA management rather than offered as isolated procedures. Rehabilitation, physical activity, load management, weight management when clinically indicated, sleep care, and treatment of relevant comorbidities remain important components of care even though their specific interaction with orthobiologic efficacy has not yet been established.

### 4.8 Limitations

Several limitations should be considered. First, although the review followed a structured integrative-review methodology with predefined eligibility criteria, thematic search domains, and documented literature-selection procedures, its broad conceptual scope required the integration of heterogeneous evidence rather than exhaustive effect estimation within each individual domain. Consequently, some relevant studies addressing specific subdomains may not have been captured, particularly where terminology and methodological approaches varied substantially across the literature.

Second, the framework integrates heterogeneous forms of evidence, including randomized controlled trials, systematic reviews, mechanistic investigations, imaging studies, biomarker research, and conceptual models. These evidence types differ substantially in methodological strength and cannot be interpreted as providing the same degree of causal support.

Third, some components of systemic soil, particularly microbiome-related factors, chronobiology, and multimodal host optimization, are supported more strongly by biological plausibility and indirect evidence than by prospective orthobiologic trials.

Fourth, the framework is primarily explanatory and has not yet been prospectively validated as a combined prediction or treatment-selection model. It remains uncertain which variables provide independent prognostic information, which are modifiable, which mediate treatment effects, and which primarily reflect disease severity.

Fifth, the evidence synthesis was centered on knee OA. Although the conceptual structure may warrant evaluation in other musculoskeletal conditions, broader applicability should not be assumed without condition-specific validation.

Finally, increasing biological stratification creates a risk of excessive complexity. A clinically useful framework could balance mechanistic detail with feasibility, reproducibility, cost, and accessibility. Future validation efforts may therefore benefit from identifying a limited set of variables that meaningfully improve treatment-response characterization, prognostic accuracy, or interpretation of clinical outcomes while preserving feasibility, reproducibility, cost-effectiveness, and accessibility.

### 4.9 Knowledge gaps and future research priorities

The framework identifies several priorities for future research, including improved patient subphenotyping, prospective reporting of systemic-soil variables, standardized characterization of orthobiologic products, validation of imaging and molecular markers, explicit assessment of pain phenotype, and adequately powered analyses of treatment-by-soil interactions.

Further priorities include preserving the distinction between BMA and BMAC, standardizing definitions of adipose-derived products, separating contextual from product-specific effects, and determining whether potentially modifiable systemic or local factors genuinely alter treatment response rather than merely correlate with outcome.

The principal knowledge gaps, their clinical and methodological implications, and corresponding research priorities for the proposed framework in knee OA are summarized in Table 4.

**TABLE 4:** Knowledge gaps and research priorities for the seed–systemic soil–local tissue soil framework in knee osteoarthritis.

Knowledge gap	Why it matters	Potential impact	Suggested research priority
Poor patient subphenotyping (Hannani et al., 2024; del Río, 2025)	Heterogeneous populations dilute biologic signal	May improve patient matching and trial interpretation	Develop phenotype/endotype-enriched study designs
Underreporting of systemic soil variables (Lana et al., 2024; Navani et al., 2025)	Host biology remains invisible in many trials	Limits explanation of interpatient variability	Standardize reporting of metabolic, sleep, and host factors
Limited integration of local tissue biology and pain phenotype (Panichi et al., 2024; Zhu et al., 2019; Salaffi et al., 2025; Herrera et al., 2024; Harkey et al., 2026)	Structure alone does not explain symptoms or response	May improve interpretation of clinical–structural dissociation	Combine imaging, pain profiling, and tissue phenotype assessment
Inconsistent distinction between BMA and BMAC (Park et al., 2025; Maruccia et al., 2026)	Marrow-derived seed is often oversimplified	Reduces seed clarity and reproducibility	Report aspiration method, harvest site, and concentration strategy
Heterogeneous adipose-derived product definitions (Batista et al., 2026)	ADSCs, SVF, and MFAT are often collapsed together	Weakens comparative inference	Enforce clear adipose product taxonomy
Lack of validated biomarkers of regenerative readiness (Boffa et al., 2021; Hannani et al., 2024)	Soil remains conceptually plausible but clinically undermeasured	Could enable biologically stratified intervention	Develop multimodal readiness biomarkers
Scarcity of multimodal trial designs (Navani et al., 2025; Hannani et al., 2024)	Single-domain outcomes miss biologic complexity	Limits mechanistic interpretation	Design trials combining clinical, imaging, and biologic endpoints
Limited long-term follow-up (Batista et al., 2026; Cao et al., 2025; Lee et al., 2019; Freitag et al., 2024; Pers et al., 2025)	Regenerative claims require temporal depth	Weakens translational confidence	Extend follow-up beyond conventional short-term horizons

## 5 Conclusion

In this integrative review, we propose a Seed–Systemic Soil–Local Tissue Soil framework to organize potential determinants of orthobiologic response heterogeneity in knee OA. The framework extends rigorous product characterization by incorporating systemic host biology and local joint and tissue characteristics. Current evidence supports substantial heterogeneity across these domains, but direct prospective evidence that modifying systemic or local soil factors alters orthobiologic efficacy remains limited. Symptomatic improvement should not be interpreted as evidence of tissue regeneration, while absence of structural regeneration does not necessarily preclude clinically meaningful symptomatic benefit. The framework should therefore be regarded as explanatory and hypothesis-generating and requires prospective validation using well-characterized products and prespecified host- and tissue-level variables.
