# Supplementary material for: Preparing the soil in knee osteoarthritis: a seed–systemic soil–local tissue soil framework for orthobiologic response heterogeneity
Source: Front Bioeng Biotechnol. 2026 Sep 8;14:1946746. doi: 10.3389/fbioe.2026.1946746 (PMC13595570; doi:10.3389/fbioe.2026.1946746)
Supplement: Supplementary file 1 [file Table1.docx]

# **Supplementary Table S1. Complete database-specific electronic search strategies**

The electronic search was conducted in June 2026 and covered publications from January 2016 through June 2026. Five complementary thematic searches were performed in each database: (1) orthobiologic products/seed; (2) systemic host biology/systemic soil; (3) local joint and tissue biology/local tissue soil; (4) imaging, biomarkers, endotypes, and stratification; and (5) contextual effects and comparator characteristics. English-language limits were applied. Human-only eligibility was applied during screening rather than as a restrictive database filter to avoid loss of reviews and incompletely indexed records. Search syntax was adapted to each database.

| Database | Search domain | Complete search strategy |
| --- | --- | --- |
| **PubMed/MEDLINE** | **S1 – Knee OA + orthobiologic products (“seed”)** | ("Osteoarthritis, Knee"[Mesh] OR "knee osteoarthritis"[tiab] OR "osteoarthritis of the knee"[tiab] OR "knee OA"[tiab]) AND ("Regenerative Medicine"[Mesh] OR orthobiologic*[tiab] OR "regenerative medicine"[tiab] OR "Platelet-Rich Plasma"[Mesh] OR "platelet-rich plasma"[tiab] OR PRP[tiab] OR "bone marrow aspirate"[tiab] OR "bone marrow aspirate concentrate"[tiab] OR BMA[tiab] OR BMAC[tiab] OR "Mesenchymal Stem Cells"[Mesh] OR "mesenchymal stromal cell*"[tiab] OR "mesenchymal stem cell*"[tiab] OR "adipose-derived stromal cell*"[tiab] OR "adipose-derived stem cell*"[tiab] OR "stromal vascular fraction"[tiab] OR SVF[tiab] OR "microfragmented adipose tissue"[tiab] OR MFAT[tiab]) AND ("2016/01/01"[Date - Publication] : "2026/06/30"[Date - Publication]) AND english[lang] |
| **PubMed/MEDLINE** | **S2 – Systemic host biology (“systemic soil”)** | ("Osteoarthritis, Knee"[Mesh] OR "knee osteoarthritis"[tiab] OR "osteoarthritis of the knee"[tiab] OR "knee OA"[tiab]) AND ("Metabolic Syndrome"[Mesh] OR "Obesity"[Mesh] OR "Insulin Resistance"[Mesh] OR "Inflammation"[Mesh] OR "Sleep"[Mesh] OR "Diet"[Mesh] OR "Exercise"[Mesh] OR "Rehabilitation"[Mesh] OR host factor*[tiab] OR metabolic health[tiab] OR metabolic syndrome[tiab] OR obes*[tiab] OR body composition[tiab] OR insulin resistance[tiab] OR glycemic control[tiab] OR systemic inflammation[tiab] OR low-grade inflammation[tiab] OR nutrition*[tiab] OR diet*[tiab] OR sleep[tiab] OR chronobiolog*[tiab] OR medication exposure[tiab] OR physical activ*[tiab] OR exercise[tiab] OR rehabilitation[tiab] OR smoking[tiab] OR psychological factor*[tiab] OR comorbid*[tiab] OR microbiome[tiab] OR microbiota[tiab]) AND ("2016/01/01"[Date - Publication] : "2026/06/30"[Date - Publication]) AND english[lang] |
| **PubMed/MEDLINE** | **S3 – Local joint/tissue biology (“local tissue soil”)** | ("Osteoarthritis, Knee"[Mesh] OR "knee osteoarthritis"[tiab] OR "osteoarthritis of the knee"[tiab] OR "knee OA"[tiab]) AND (synovitis[tiab] OR effusion[tiab] OR cartilage[tiab] OR "subchondral bone"[tiab] OR "bone marrow lesion*"[tiab] OR menisc*[tiab] OR "meniscal extrusion"[tiab] OR "mechanical alignment"[tiab] OR malalignment[tiab] OR "infrapatellar fat pad"[tiab] OR "Hoffa fat pad"[tiab] OR "periarticular muscle*"[tiab] OR "joint phenotype"[tiab] OR "tissue phenotype"[tiab] OR nocicept*[tiab] OR "neuropathic-like pain"[tiab] OR "pain sensitization"[tiab] OR "central sensitization"[tiab]) AND ("2016/01/01"[Date - Publication] : "2026/06/30"[Date - Publication]) AND english[lang] |
| **PubMed/MEDLINE** | **S4 – Imaging, biomarkers, endotypes and stratification** | ("Osteoarthritis, Knee"[Mesh] OR "knee osteoarthritis"[tiab] OR "osteoarthritis of the knee"[tiab] OR "knee OA"[tiab]) AND ("Magnetic Resonance Imaging"[Mesh] OR MRI[tiab] OR "magnetic resonance imaging"[tiab] OR biomarker*[tiab] OR "Biomarkers"[Mesh] OR "imaging biomarker*"[tiab] OR "biochemical marker*"[tiab] OR "synovial fluid"[tiab] OR endotype*[tiab] OR phenotype*[tiab] OR "molecular endotype*"[tiab] OR "prognostic factor*"[tiab] OR "predictive factor*"[tiab] OR "treatment response"[tiab] OR responder*[tiab] OR "patient stratification"[tiab] OR "precision medicine"[tiab] OR "treatment effect modifier*"[tiab]) AND ("2016/01/01"[Date - Publication] : "2026/06/30"[Date - Publication]) AND english[lang] |
| **PubMed/MEDLINE** | **S5 – Contextual effects and comparator characteristics** | ("Osteoarthritis, Knee"[Mesh] OR "knee osteoarthritis"[tiab] OR "osteoarthritis of the knee"[tiab] OR "knee OA"[tiab]) AND ("Injections, Intra-Articular"[Mesh] OR intra-articular[tiab] OR injection*[tiab] OR orthobiologic*[tiab] OR biologic*[tiab]) AND ("Placebo Effect"[Mesh] OR contextual effect*[tiab] OR placebo effect*[tiab] OR placebo response[tiab] OR saline injection*[tiab] OR sham[tiab] OR active comparator*[tiab] OR corticosteroid*[tiab] OR hyaluronic acid[tiab] OR platelet-rich plasma[tiab] OR rehabilitation[tiab] OR within-group[tiab] OR between-group[tiab] OR "treatment effect"[tiab]) AND ("2016/01/01"[Date - Publication] : "2026/06/30"[Date - Publication]) AND english[lang] |
| **Embase (Elsevier/Embase.com)** | **S1 – Knee OA + orthobiologic products (“seed”)** | ('knee osteoarthritis'/exp OR 'knee osteoarthritis':ti,ab,kw OR 'osteoarthritis of the knee':ti,ab,kw OR 'knee oa':ti,ab,kw) AND ('regenerative medicine'/exp OR orthobiologic*:ti,ab,kw OR 'regenerative medicine':ti,ab,kw OR 'platelet rich plasma'/exp OR 'platelet-rich plasma':ti,ab,kw OR prp:ti,ab,kw OR 'bone marrow aspirate':ti,ab,kw OR 'bone marrow aspirate concentrate':ti,ab,kw OR bma:ti,ab,kw OR bmac:ti,ab,kw OR 'mesenchymal stem cell'/exp OR 'mesenchymal stromal cell*':ti,ab,kw OR 'mesenchymal stem cell*':ti,ab,kw OR 'adipose-derived stromal cell*':ti,ab,kw OR 'adipose-derived stem cell*':ti,ab,kw OR 'stromal vascular fraction':ti,ab,kw OR svf:ti,ab,kw OR 'microfragmented adipose tissue':ti,ab,kw OR mfat:ti,ab,kw) AND [2016-2026]/py AND [english]/lim |
| **Embase (Elsevier/Embase.com)** | **S2 – Systemic host biology (“systemic soil”)** | ('knee osteoarthritis'/exp OR 'knee osteoarthritis':ti,ab,kw OR 'osteoarthritis of the knee':ti,ab,kw OR 'knee oa':ti,ab,kw) AND ('metabolic syndrome'/exp OR obesity/exp OR 'insulin resistance'/exp OR inflammation/exp OR sleep/exp OR diet/exp OR exercise/exp OR rehabilitation/exp OR 'host factor*':ti,ab,kw OR 'metabolic health':ti,ab,kw OR 'metabolic syndrome':ti,ab,kw OR obes*:ti,ab,kw OR 'body composition':ti,ab,kw OR 'insulin resistance':ti,ab,kw OR 'glycemic control':ti,ab,kw OR 'systemic inflammation':ti,ab,kw OR 'low-grade inflammation':ti,ab,kw OR nutrition*:ti,ab,kw OR diet*:ti,ab,kw OR sleep:ti,ab,kw OR chronobiolog*:ti,ab,kw OR 'medication exposure':ti,ab,kw OR 'physical activity':ti,ab,kw OR exercise:ti,ab,kw OR rehabilitation:ti,ab,kw OR smoking:ti,ab,kw OR 'psychological factor*':ti,ab,kw OR comorbid*:ti,ab,kw OR microbiome:ti,ab,kw OR microbiota:ti,ab,kw) AND [2016-2026]/py AND [english]/lim |
| **Embase (Elsevier/Embase.com)** | **S3 – Local joint/tissue biology (“local tissue soil”)** | ('knee osteoarthritis'/exp OR 'knee osteoarthritis':ti,ab,kw OR 'osteoarthritis of the knee':ti,ab,kw OR 'knee oa':ti,ab,kw) AND (synovitis:ti,ab,kw OR effusion:ti,ab,kw OR cartilage:ti,ab,kw OR 'subchondral bone':ti,ab,kw OR 'bone marrow lesion*':ti,ab,kw OR menisc*:ti,ab,kw OR 'meniscal extrusion':ti,ab,kw OR 'mechanical alignment':ti,ab,kw OR malalignment:ti,ab,kw OR 'infrapatellar fat pad':ti,ab,kw OR 'hoffa fat pad':ti,ab,kw OR 'periarticular muscle*':ti,ab,kw OR 'joint phenotype':ti,ab,kw OR 'tissue phenotype':ti,ab,kw OR nocicept*:ti,ab,kw OR 'neuropathic-like pain':ti,ab,kw OR 'pain sensitization':ti,ab,kw OR 'central sensitization':ti,ab,kw) AND [2016-2026]/py AND [english]/lim |
| **Embase (Elsevier/Embase.com)** | **S4 – Imaging, biomarkers, endotypes and stratification** | ('knee osteoarthritis'/exp OR 'knee osteoarthritis':ti,ab,kw OR 'osteoarthritis of the knee':ti,ab,kw OR 'knee oa':ti,ab,kw) AND ('magnetic resonance imaging'/exp OR mri:ti,ab,kw OR 'magnetic resonance imaging':ti,ab,kw OR biomarker*:ti,ab,kw OR 'imaging biomarker*':ti,ab,kw OR 'biochemical marker*':ti,ab,kw OR 'synovial fluid':ti,ab,kw OR endotype*:ti,ab,kw OR phenotype*:ti,ab,kw OR 'molecular endotype*':ti,ab,kw OR 'prognostic factor*':ti,ab,kw OR 'predictive factor*':ti,ab,kw OR 'treatment response':ti,ab,kw OR responder*:ti,ab,kw OR 'patient stratification':ti,ab,kw OR 'precision medicine':ti,ab,kw OR 'treatment effect modifier*':ti,ab,kw) AND [2016-2026]/py AND [english]/lim |
| **Embase (Elsevier/Embase.com)** | **S5 – Contextual effects and comparator characteristics** | ('knee osteoarthritis'/exp OR 'knee osteoarthritis':ti,ab,kw OR 'osteoarthritis of the knee':ti,ab,kw OR 'knee oa':ti,ab,kw) AND ('intraarticular drug administration'/exp OR 'intra-articular':ti,ab,kw OR injection*:ti,ab,kw OR orthobiologic*:ti,ab,kw OR biologic*:ti,ab,kw) AND ('placebo effect'/exp OR 'contextual effect*':ti,ab,kw OR 'placebo effect*':ti,ab,kw OR 'placebo response':ti,ab,kw OR 'saline injection*':ti,ab,kw OR sham:ti,ab,kw OR 'active comparator*':ti,ab,kw OR corticosteroid*:ti,ab,kw OR 'hyaluronic acid':ti,ab,kw OR 'platelet-rich plasma':ti,ab,kw OR rehabilitation:ti,ab,kw OR 'within-group':ti,ab,kw OR 'between-group':ti,ab,kw OR 'treatment effect':ti,ab,kw) AND [2016-2026]/py AND [english]/lim |
| **Scopus** | **S1 – Knee OA + orthobiologic products (“seed”)** | TITLE-ABS-KEY("knee osteoarthritis" OR "osteoarthritis of the knee" OR "knee OA") AND TITLE-ABS-KEY(orthobiologic* OR "regenerative medicine" OR "platelet-rich plasma" OR PRP OR "bone marrow aspirate" OR "bone marrow aspirate concentrate" OR BMA OR BMAC OR "mesenchymal stromal cell*" OR "mesenchymal stem cell*" OR "adipose-derived stromal cell*" OR "adipose-derived stem cell*" OR "stromal vascular fraction" OR SVF OR "microfragmented adipose tissue" OR MFAT) AND PUBYEAR > 2015 AND PUBYEAR < 2027 |
| **Scopus** | **S2 – Systemic host biology (“systemic soil”)** | TITLE-ABS-KEY("knee osteoarthritis" OR "osteoarthritis of the knee" OR "knee OA") AND TITLE-ABS-KEY("host factor*" OR "metabolic health" OR "metabolic syndrome" OR obes* OR "body composition" OR "insulin resistance" OR "glycemic control" OR "systemic inflammation" OR "low-grade inflammation" OR nutrition* OR diet* OR sleep OR chronobiolog* OR "medication exposure" OR "physical activity" OR exercise OR rehabilitation OR smoking OR "psychological factor*" OR comorbid* OR microbiome OR microbiota) AND PUBYEAR > 2015 AND PUBYEAR < 2027 |
| **Scopus** | **S3 – Local joint/tissue biology (“local tissue soil”)** | TITLE-ABS-KEY("knee osteoarthritis" OR "osteoarthritis of the knee" OR "knee OA") AND TITLE-ABS-KEY(synovitis OR effusion OR cartilage OR "subchondral bone" OR "bone marrow lesion*" OR menisc* OR "meniscal extrusion" OR "mechanical alignment" OR malalignment OR "infrapatellar fat pad" OR "Hoffa fat pad" OR "periarticular muscle*" OR "joint phenotype" OR "tissue phenotype" OR nocicept* OR "neuropathic-like pain" OR "pain sensitization" OR "central sensitization") AND PUBYEAR > 2015 AND PUBYEAR < 2027 |
| **Scopus** | **S4 – Imaging, biomarkers, endotypes and stratification** | TITLE-ABS-KEY("knee osteoarthritis" OR "osteoarthritis of the knee" OR "knee OA") AND TITLE-ABS-KEY(MRI OR "magnetic resonance imaging" OR biomarker* OR "imaging biomarker*" OR "biochemical marker*" OR "synovial fluid" OR endotype* OR phenotype* OR "molecular endotype*" OR "prognostic factor*" OR "predictive factor*" OR "treatment response" OR responder* OR "patient stratification" OR "precision medicine" OR "treatment effect modifier*") AND PUBYEAR > 2015 AND PUBYEAR < 2027 |
| **Scopus** | **S5 – Contextual effects and comparator characteristics** | TITLE-ABS-KEY("knee osteoarthritis" OR "osteoarthritis of the knee" OR "knee OA") AND TITLE-ABS-KEY("intra-articular" OR injection* OR orthobiologic* OR biologic*) AND TITLE-ABS-KEY("contextual effect*" OR "placebo effect*" OR "placebo response" OR "saline injection*" OR sham OR "active comparator*" OR corticosteroid* OR "hyaluronic acid" OR "platelet-rich plasma" OR rehabilitation OR "within-group" OR "between-group" OR "treatment effect") AND PUBYEAR > 2015 AND PUBYEAR < 2027 |
| **Web of Science Core Collection** | **S1 – Knee OA + orthobiologic products (“seed”)** | TS=("knee osteoarthritis" OR "osteoarthritis of the knee" OR "knee OA") AND TS=(orthobiologic* OR "regenerative medicine" OR "platelet-rich plasma" OR PRP OR "bone marrow aspirate" OR "bone marrow aspirate concentrate" OR BMA OR BMAC OR "mesenchymal stromal cell*" OR "mesenchymal stem cell*" OR "adipose-derived stromal cell*" OR "adipose-derived stem cell*" OR "stromal vascular fraction" OR SVF OR "microfragmented adipose tissue" OR MFAT) AND PY=(2016-2026) |
| **Web of Science Core Collection** | **S2 – Systemic host biology (“systemic soil”)** | TS=("knee osteoarthritis" OR "osteoarthritis of the knee" OR "knee OA") AND TS=("host factor*" OR "metabolic health" OR "metabolic syndrome" OR obes* OR "body composition" OR "insulin resistance" OR "glycemic control" OR "systemic inflammation" OR "low-grade inflammation" OR nutrition* OR diet* OR sleep OR chronobiolog* OR "medication exposure" OR "physical activity" OR exercise OR rehabilitation OR smoking OR "psychological factor*" OR comorbid* OR microbiome OR microbiota) AND PY=(2016-2026) |
| **Web of Science Core Collection** | **S3 – Local joint/tissue biology (“local tissue soil”)** | TS=("knee osteoarthritis" OR "osteoarthritis of the knee" OR "knee OA") AND TS=(synovitis OR effusion OR cartilage OR "subchondral bone" OR "bone marrow lesion*" OR menisc* OR "meniscal extrusion" OR "mechanical alignment" OR malalignment OR "infrapatellar fat pad" OR "Hoffa fat pad" OR "periarticular muscle*" OR "joint phenotype" OR "tissue phenotype" OR nocicept* OR "neuropathic-like pain" OR "pain sensitization" OR "central sensitization") AND PY=(2016-2026) |
| **Web of Science Core Collection** | **S4 – Imaging, biomarkers, endotypes and stratification** | TS=("knee osteoarthritis" OR "osteoarthritis of the knee" OR "knee OA") AND TS=(MRI OR "magnetic resonance imaging" OR biomarker* OR "imaging biomarker*" OR "biochemical marker*" OR "synovial fluid" OR endotype* OR phenotype* OR "molecular endotype*" OR "prognostic factor*" OR "predictive factor*" OR "treatment response" OR responder* OR "patient stratification" OR "precision medicine" OR "treatment effect modifier*") AND PY=(2016-2026) |
| **Web of Science Core Collection** | **S5 – Contextual effects and comparator characteristics** | TS=("knee osteoarthritis" OR "osteoarthritis of the knee" OR "knee OA") AND TS=("intra-articular" OR injection* OR orthobiologic* OR biologic*) AND TS=("contextual effect*" OR "placebo effect*" OR "placebo response" OR "saline injection*" OR sham OR "active comparator*" OR corticosteroid* OR "hyaluronic acid" OR "platelet-rich plasma" OR rehabilitation OR "within-group" OR "between-group" OR "treatment effect") AND PY=(2016-2026) |

**Search limits and additional procedures:** PubMed/MEDLINE searches incorporated English-language and publication-date limits directly into the electronic strategy. Equivalent date and language limits were applied in Embase. For Scopus and Web of Science, publication-year restrictions were incorporated in the search and English-language filtering was applied through the database interface. Because the searches were conducted in June 2026, the 2026 component included records available up to the date of search. Human eligibility was assessed during title/abstract and full-text screening rather than through a universal human database filter. Records generated by the five complementary searches were combined and deduplicated within each database before the four database libraries were merged and deduplicated across databases. Seminal publications predating 2016 and selected mechanistic preclinical studies were eligible only through targeted citation tracking and were classified separately from the contemporary human evidence base.
