# Supplementary material for: Preparing the soil in knee osteoarthritis: a seed–systemic soil–local tissue soil framework for orthobiologic response heterogeneity
Source: Front Bioeng Biotechnol. 2026 Sep 8;14:1946746. doi: 10.3389/fbioe.2026.1946746 (PMC13595570; doi:10.3389/fbioe.2026.1946746)
Supplement: Supplementary file 2 [file Table2.docx]

# **Supplementary Table S2. Literature identification and selection process**

| Stage of literature identification and selection | Records/publications, n |
| --- | --- |
| Records retrieved from PubMed/MEDLINE | 801 |
| Records retrieved from Embase | 1,017 |
| Records retrieved from Scopus | 1,264 |
| Records retrieved from Web of Science Core Collection | 932 |
| **Total database records identified** | **4,014** |
| Duplicate records removed after combining database libraries | 1,726 |
| **Unique records remaining after deduplication** | **2,288** |
| Records screened by title and abstract | 2,288 |
| Records excluded during title/abstract screening | 2,142 |
| **Full-text publications assessed for eligibility** | **146** |
| Full-text publications excluded | 124 |
| **Publications retained from the electronic database search** | **22** |
| Additional potentially relevant publications identified through backward/forward citation tracking | 11 |
| Additional publications excluded after full-text assessment/citation review | 7 |
| **Additional publications included through citation tracking** | **4** |
| **Total publications included in the integrative synthesis** | **26** |

**Notes:** Records retrieved from each database represent the unique yield after combining and internally deduplicating the five thematic searches performed within that database. Cross-database duplicate removal was subsequently performed after merging the four database libraries. The 22 publications retained from the primary electronic search constituted the contemporary evidence base. Four additional publications were incorporated through targeted citation tracking, including seminal or mechanistically relevant sources that met the predefined exceptions described in the eligibility criteria. Citation-tracking publications were assessed against the same conceptual relevance criteria but were not considered part of the primary 2016–2026 electronic search yield.
