# Supplementary material for: Preparing the soil in knee osteoarthritis: a seed–systemic soil–local tissue soil framework for orthobiologic response heterogeneity
Source: Front Bioeng Biotechnol. 2026 Sep 8;14:1946746. doi: 10.3389/fbioe.2026.1946746 (PMC13595570; doi:10.3389/fbioe.2026.1946746)
Supplement: Supplementary file 3 [file Table3.docx]

**Supplementary Table S3. Evidence-status mapping of the principal domains and propositions of the Seed–Systemic Soil–Local Tissue Soil framework**

| **Framework domain** | **Topic / proposition evaluated** | **Main evidence type** | **Representative evidence** | **Evidence status** | **Current evidentiary role** | **Interpretation within the framework** |
| --- | --- | --- | --- | --- | --- | --- |
| **Seed** | Orthobiologic products within the same nominal category may differ substantially in composition and biological characteristics | Human clinical literature; contemporary reviews | Andia et al., 2025; Navani et al., 2025 | Direct evidence-supported observation | Direct product-characterization evidence; not a treatment-effect-modifier claim | Product labels alone may inadequately characterize the biological intervention; source, processing, composition, dose, and delivery require explicit characterization. |
| **Seed** | ADSCs, SVF, and MFAT should not be considered biologically interchangeable | Systematic review of RCTs; individual RCTs | Batista et al., 2026; Lee et al., 2019; Freitag et al., 2024; Pers et al., 2025 | Direct evidence-supported observation | Direct product-category distinction; treatment-response implications vary across preparations | Adipose-derived interventions differ in processing, cellular composition, dose, and clinical protocols and should be reported as distinct product categories. |
| **Seed** | MSC-based interventions can produce symptomatic benefit in knee OA, but effects vary across products and trials | Systematic review and meta-analysis of RCTs | Cao et al., 2025 | Direct evidence-supported observation | Clinical-effect evidence at class level; heterogeneity limits prediction for individual products or phenotypes | Clinical activity is supported in part of the literature, but MSC-based interventions should not be interpreted as a uniform treatment category. |
| **Seed** | BMA and BMAC characteristics are influenced by harvesting and processing procedures | Human technical/clinical evidence; narrative synthesis | Park et al., 2025; Maruccia et al., 2026 | Translationally supported inference | Product-characterization and mechanistic relevance; causal impact on clinical outcome remains unproven | Aspiration technique, harvest site, blood contamination, and processing may alter product composition and should be documented. |
| **Systemic soil** | Metabolic-inflammatory status may contribute to heterogeneity in biological responsiveness | Human OA literature; contemporary reviews | Tang et al., 2025; Lana et al., 2024; Navani et al., 2025 | Translationally supported inference | Association/prognostic relevance; orthobiologic treatment-effect modification and causal benefit of optimization remain unproven | Obesity, metabolic dysfunction, insulin resistance, and low-grade inflammation are plausible host-level stratification variables, but direct evidence that their modification improves orthobiologic efficacy is insufficient. |
| **Systemic soil** | Sleep and chronobiology may contribute to host biological readiness | Host-oriented conceptual and human associative evidence | Lana et al., 2024 | Hypothesis-generating proposition | Association/biological plausibility; treatment-effect modification and causal benefit of sleep optimization are unproven | Sleep is relevant to pain, recovery, and neuroimmune regulation, but should not be considered a validated prerequisite or enhancer of orthobiologic treatment. |
| **Systemic soil** | Microbiome-related mechanisms may contribute to systemic biological readiness | Translational and associative literature | Longo et al., 2024 | Hypothesis-generating proposition | Mechanistic plausibility and association; no validated prognostic, treatment-modifying, or causal optimization role | Gut–joint interactions are biologically plausible, but microbiome-directed strategies remain exploratory for orthobiologic stratification. |
| **Systemic soil** | Multidomain host optimization may improve regenerative treatment responsiveness | Conceptual framework | Lana et al., 2024 | Hypothesis-generating proposition | Conceptual therapeutic hypothesis; causal benefit of bundled optimization has not been demonstrated | SDIMMMER provides a useful host-oriented structure, but prospective evidence that the complete optimization package enhances orthobiologic efficacy is unavailable. |
| **Local tissue soil** | Synovitis is a biologically relevant component of the local OA environment | Human OA inflammatory and imaging literature | Panichi et al., 2024; Herrera et al., 2024 | Translationally supported inference | Association/prognostic candidate; orthobiologic treatment-effect modification remains unproven | Synovial inflammatory activity may contribute to local tissue receptivity and is a feasible candidate variable for prospective stratification. |
| **Local tissue soil** | Subchondral remodeling may contribute to sensory amplification and OA pain | Mechanistic/preclinical evidence | Zhu et al., 2019 | Translationally supported inference | Mechanistic plausibility; no established prognostic or orthobiologic treatment-modifying role | Subchondral bone provides a plausible pathway linking tissue remodeling to nociceptive activity; extrapolation to orthobiologic response requires validation. |
| **Cross-cutting modifier** | Neuropathic-like symptoms, catastrophizing, and central sensitization contribute to symptom heterogeneity | Human observational evidence | Salaffi et al., 2025 | Translationally supported inference | Association/prognostic and stratification candidate; treatment-by-pain-phenotype interaction remains unproven | Pain phenotype may contribute to symptom–structure discordance and should be treated as a cross-cutting modifier rather than a strictly local tissue characteristic. |
| **Local tissue soil / Imaging** | MRI can characterize multiple structural and inflammatory components of the OA joint beyond cartilage alone | Human imaging literature; imaging reviews | Herrera et al., 2024; Harkey et al., 2026 | Direct evidence-supported observation | Direct phenotypic/structural characterization; not by itself evidence of treatment-effect modification | MRI can characterize synovitis, bone marrow lesions, meniscal pathology, subchondral changes, fat-pad abnormalities, and other local-tissue features. |
| **Local tissue soil / Imaging** | Baseline MRI characteristics may predict response to specific orthobiologic interventions | Imaging/prognostic literature | Herrera et al., 2024; Harkey et al., 2026 | Translationally supported inference | Prognostic/predictive candidate; treatment-specific effect modification remains incompletely validated | Imaging is promising for trial enrichment and stratification, but treatment-specific predictive validity remains insufficient for routine selection. |
| **Biomarkers / Endotypes** | Biochemical and synovial-fluid biomarkers characterize inflammatory and matrix-related OA processes | Systematic review; biomarker reviews | Boffa et al., 2021; Hannani et al., 2024 | Direct evidence-supported observation | Direct biological/phenotypic association; no single validated regenerative-readiness marker | Multiple biomarkers reflect OA-related biological processes, but no routine-ready marker defines regenerative readiness. |
| **Biomarkers / Endotypes** | Biomarkers or molecular endotypes may support prediction or stratification of orthobiologic response | Biomarker/endotype literature | Boffa et al., 2021; Hannani et al., 2024 | Translationally supported inference | Prognostic/stratification candidate; treatment-effect modification and causal utility remain unproven | Biomarkers are currently more appropriate for research and trial enrichment than for routine clinical selection of orthobiologic therapy. |
| **Cross-cutting** | Contextual and nonspecific procedural effects contribute to symptomatic improvement after intra-articular cell-based interventions | Systematic review and meta-analysis of RCTs | Yin et al., 2025 | Direct evidence-supported observation | Direct evidence of contribution to total observed response; not evidence of biological product inactivity | Total clinical improvement cannot automatically be attributed to the biological product itself. |
| **Cross-cutting** | Active comparators can produce clinically meaningful improvement and influence between-group effect estimates | RCT; umbrella review; network meta-analyses | Mautner et al., 2023; Qiao et al., 2023; Jawanda et al., 2024; Bruyère et al., 2025 | Direct evidence-supported observation | Direct comparator-effect evidence; influences interpretation of incremental product-specific efficacy | Failure to demonstrate superiority over an active comparator should not be interpreted equivalently to failure against an inert control. |
| **Disease stage / Stratification** | Earlier or pre-OA biological states may represent more receptive targets for regenerative intervention | Conceptual/emerging evidence | del Río, 2025 | Hypothesis-generating proposition | Biological plausibility/stratification hypothesis; prognostic and treatment-modifying roles are unproven | Earlier biological vulnerability may represent a future stratification opportunity, but improved treatment response has not been prospectively demonstrated. |
| **Integrated framework** | Orthobiologic response may reflect interactions among seed characteristics, systemic host biology, and local tissue receptivity | Integrative conceptual synthesis across evidence domains | Batista et al., 2026; Tang et al., 2025; Lana et al., 2024; Navani et al., 2025; Andia et al., 2025; Boffa et al., 2021; Hannani et al., 2024 | Hypothesis-generating proposition | Hypothesis-generating treatment-effect-modification model; requires prospective interaction testing | The three-layer interaction is the central explanatory proposition of the framework and requires prospective testing of treatment-by-host and treatment-by-tissue interactions. |

**Note.** Evidence status follows the three-level interpretive framework described in Section 2.7. The 'Current evidentiary role' column distinguishes association/prognostic relevance from treatment-effect modification, mediation, and causal benefit of factor modification; association or prognostic relevance was not interpreted as proof of effect modification or causal benefit. This classification is interpretive and does not constitute a formal GRADE assessment.
